# Experiences of COVID-19-related anti-Asian discrimination and affective reactions in a multiple race sample of U.S. young adults

**DOI:** 10.1186/s12889-021-11559-1

**Published:** 2021-08-18

**Authors:** Hyeouk Chris Hahm, Casey D. Xavier Hall, Kana Tsurudome Garcia, Anna Cavallino, Yoonsook Ha, Yvette C. Cozier, Cindy Liu

**Affiliations:** 1 Boston University School of Social Work, 264 Bay State Road, Boston, MA 02215 USA; 2 Northwestern University’s Institute for Sexual and Gender Minority Health and Wellbeing, 625 N. Michigan Avenue, Suite 1400, Chicago, IL 60611 USA; 3 Boston University School of Public Health, 715 Albany Street, Boston, MA 02118 USA; 4 Brigham and Women’s Hospital, 75 Francis St, Boston, MA 02115 USA

**Keywords:** Discrimination, Racism, Anti-Asian discrimination, COVID-19, China virus, Xenophobia

## Abstract

**Background:**

Little remains known about both Asian and Asian American (A/AA) and non-Asian young adults’ experiences and affective reactions regarding COVID-19 anti-Asian discrimination. To our knowledge, this is the first study that explores the nature and impact of COVID-19 anti-Asian discrimination within a multi-racial sample.

**Methods:**

This study uses qualitative open-ended responses from a sub-sample of Wave I of the COVID-19 Adult Resilience Experiences Study (CARES) data collected between March to September 2020. Thematic analysis was used to explore two open-ended questions: “Are there experiences we missed in the survey so far that you wish to describe?” and “What are your thoughts about the current social climate?” The data analysis for this study focused on 113 discrimination or racism-related comments.

**Results:**

A total of 1331 young adults completed an online survey of which 611 provided comments; a multi-racial sample of 95 individuals (65.3% non-Asians, 24.7% A/AA) contributed 113 COVID-19 anti-Asian discrimination or racism-related comments. Two overarching themes were: types of discrimination (societal, interpersonal, intrapersonal) and affective reactions to discrimination (fear, anxiety/distress, hopelessness/depression, and avoidance). Not only did both A/AA and non-Asian participants report witnessing or hearing reports of anti-Asian discrimination, but both groups described having negative affective reactions to anti-Asian discrimination.

**Conclusion:**

Anti-Asian discrimination in the face of COVID may be more widespread than initial reports indicate. Our finding suggests that anti-Asian discrimination is a societal illness that impacts all populations in the U.S. This calls for cross-racial coalitions and solidarity in the fight against discrimination and racism.

**Supplementary Information:**

The online version contains supplementary material available at 10.1186/s12889-021-11559-1.

## Background

The emergence of the coronavirus (COVID-19) in December of 2019 from Wuhan, China has led to a global health crisis. As of June 2021, the COVID-19 death toll has surpassed 3.8 million worldwide, with over 616,000 deaths within the U.S. [1]. The COVID-19 pandemic has threatened the well-being of individuals due to its global health and economic impact. Similar to other outbreaks throughout history [2], the COVID-19 pandemic has resulted in the blaming of individuals on the basis of race/ethnicity and national origin. There have been increasing COVID-19-related discrimination incidents in the U.S. against Asians and Asian Americans (A/AA), given the origin of the virus [3]. In March 2021, STOP AAPI Hate Report received a surge of reported hate incidents from 3795 to 6603 in the same month that six Asian women were shot and killed in Atlanta [4]. A/AA have experienced in-person and overt discrimination in the form of verbal and/or physical assaults, including racial slurs, wrongful workplace terminations, or being stared at. A/AA communities have countered discrimination through campaigns such as #IAmNotAVirus; however, these types of experiences may lead to the internalization of racist attitudes and stereotypes [5].

The most notable anti-Asian COVID-19-related discrimination has stemmed from the former president of the U.S., referring to the virus as the “China virus” or “Kung Flu” [6]. President Trump made remarks about the origins of the virus, including saying “It’s China’s fault” as early as March 2020, and continued to politicize and weaponize this language for the remainder of his presidency even after news of pandemic-related anti-Asian attacks surfaced [7, 8]. In a study following Twitter, researchers collected 2.7 million related tweets five hours before President Trump’s announcement of his and the First Lady’s COVID-19 diagnosis on Oct. 2, lasting until Oct. 5 [8]. In this sample, anti-Asian tweets and conspiracy theory tweets regarding the origins of COVID-19 increased by 85%, with roughly 60% of the related tweets being anti-Asian rhetoric [8]. The purposeful and persistent use of racist terminology, even after the request by the World Health Organization (WHO) for individuals to refrain from its use, is highly stigmatizing. These public statements may intensify racism against A/AA [8] as suggested by public leaders and the latest research findings (e.g., U.S. senators).

Social media platforms such as Twitter, Facebook, and Instagram have further engaged in virtual displays of racism against A/AAs. Data gathered between November 1, 2019, to March 22, 2020, from two prominent online web platforms revealed significant increases in racial slurs such as “#WuhanVirus,” “#Kung-Flu,” “#Chinakidsstayhome,” and “#ChingChong.” The term “Chink” was the most popular slur, increasing from about 1250 Twitter mentions to more than 3500 mentions during March 2020 alone [3, 9].

While the negative health impacts of discrimination on A/AA are documented in the literature [10–12], the impacts of COVID-19-related anti-Asian racial discrimination on non-Asian communities have not been examined. The Harrell Model (2000) posits physical, psychological, social, functional, and spiritual consequences of discrimination [13]. In fact, this model and prior research also suggest that vicarious experiences of racism can lead to distress, anxiety, heightened sense of danger, and other psychological reactions irrespective of the racial identity of the indirect target of these experiences [13, 14]. In other words, COVID-19-related anti-Asian discrimination that encompasses directly witnessing, viewing online, or the relaying of personal narratives, may have negative impacts on the mental health of both A/AA and non-Asian people. Thus the vicarious ripple effect of discrimination due to the COVID-19 pandemic indicates that the consequences not only affect the A/AA who directly experienced discrimination, but likely extends to other non-Asian people as well. As such, the impact of this rapid increase in discrimination may be much greater and far reaching than the numbers of attacks reported in the news. Indeed, since the COVID-19 pandemic, many non-Asian Americans have voiced their concerns after witnessing their friends, family members, or even strangers deal with anti-Asian discrimination. However, most studies of pandemic discrimination focus on A/AA people who directly experience discrimination rather than the broader community who may experience the discrimination vicariously.

A growing number of studies have begun to investigate the impact of discrimination on Asian American families [15], Asian American young adults [16], or Asians in Asian countries [17]. Studies have also highlighted the increasing impact of social media on this type of discrimination [18, 19]. However, very few studies have yet to provide empirical data documenting the impact of COVID-19-related discrimination on non-Asians in the U.S.

### The current study

The current study seeks to examine qualitative descriptions of COVID-19-related discrimination experiences among A/AA and non-Asian young adults from the U.S. through the COVID-19 Adult Resilience Experiences Study (CARES) [20]. We seek to explore two research questions: 1) What types of discrimination did U.S. young adults, across races, experience during the COVID-19 pandemic? And 2) What type(s) of affective reactions were elicited by these discriminatory experiences? We used thematic analysis of open-ended survey responses. To our best knowledge, this is the first study to investigate the impacts of COVID-19 anti-Asian discrimination in a multi-race sample, including A/AA and non-Asian young adults in the U.S.

## Methods

### Data

This study uses data from Wave I (April through September 2020) of the COVID-19-CARES [21], a longitudinal study examining the psychosocial experiences of individuals ages 18–30 years across three time points from 2020 to 2021. The purpose of CARES is to assess a range of COVID-19-related experiences, including discrimination, psychiatric symptoms, and physical and mental health functioning among young adults. Subjects were recruited through social media, email listservs, and word of mouth to complete a 30-min online survey (Supplementary File 1) which included two open-ended qualitative response questions: 1) “Are there experiences we missed in the survey so far that you wish to describe?,” and 2) “What are your thoughts about the current social climate?”

All participants were either currently living in the U.S. or attending U.S.-based educational institutions. To ensure data quality, the online survey embedded various attention checks and human verification questions. Every 10th participant who enrolled received a $25 gift card. This study was reviewed and approved by the Boston University Institutional Review Board (IRB). The current analysis is focused on the subset of study participants who responded to the open-ended qualitative response questions collected during Wave I of the study.

### Analysis

**Fig. 1:**
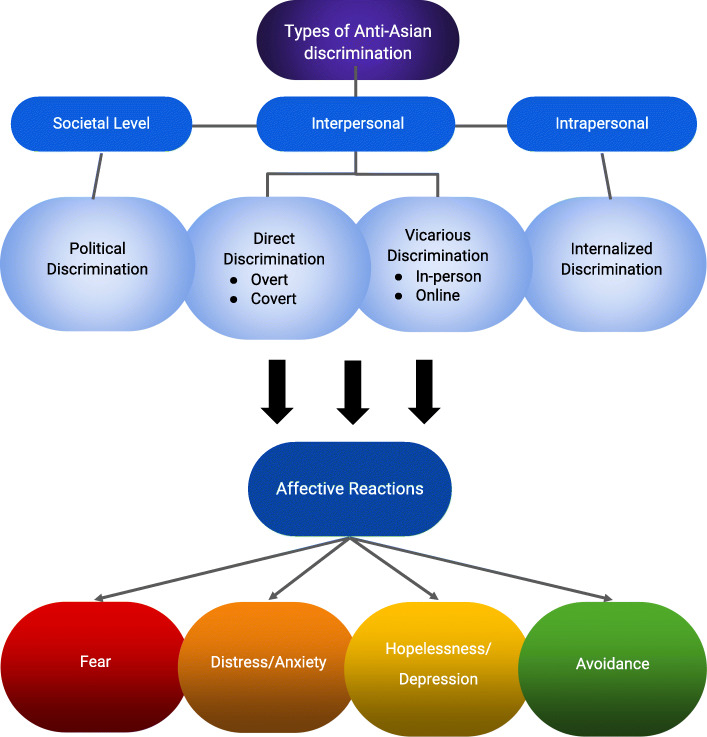
Conceptualizing Types of Discrimination and Affective Reactions (*n* = 113)

Our analysis focused on open-ended responses to open-ended questions, and the terms racism and discrimination were used interchangeably because participants tended not to make specific distinctions. Qualitative analyses were conducted by three members of the research team (HCH, KG, AC) who independently read and sorted the comments. Out of 1331 participants, 611 participants provided a response for two open-ended survey questions. “Are there experiences we missed in the survey so far that you wish to describe?” and “What are your thoughts about the current social climate?” into four major themes: discrimination/racism (*n* = 113), college relocation (*n* = 167), political climate (*n* = 54), family (*n* = 32), work (*n* = 20), relationships (*n* = 12) as well as other concerns. Discrimination was the second most common theme among the five themes that were discovered based on 611 responses. Given our goal to examine discrimination- or racism-related content, we restricted the analysis to discrimination-related comments (*n* = 113), reported by 95 respondents. We organized and analyzed the open-ended survey response data into a matrix-based system according to themes [22], following the five stages of thematic analysis--the Framework Methods [23]. First, each qualitative research team member read the data independently. Second, each member read the text, line by line, and coded the responses describing discrimination-related comments. Third, the team members worked together to develop an analytic framework. They compared individual codes and agreed on the standardized set of codes to be applied to all subsequent comments. Any differences in individual coding were reconciled through a group meeting, in which the standardized set of codes were created as a whole, providing reasoning for each code to ensure intercoder reliability. Codes were then grouped into categories resulting in the development of a tree diagram. Fourth, this analytic framework was then applied to the rest of the responses, whereby a spreadsheet was used to generate a matrix. The data was then charted into the matrix by summarizing the categorized data from each transcript. Finally, team members identified the characteristics and differences within the data to discover theoretical concepts and map the connections between the categories. From this, the following themes were identified: types of discrimination, manifestations of discrimination (direct experience or vicarious), and reactions to discrimination (Fig. 1). Reliability among the coders was maintained through a consensus approach of comparing codes across the same data by multiple members of the research team in order to resolve ambiguities. Resolution was achieved through the agreement of a standardized code that encompassed all three coders’ findings.

## Results

Between April 13 and September 30, 2020, a total of 1331 young adults completed the 30-min Wave I CARES online survey (Supplementary File 1). Of this, 611 participants responded to at least one of the two open-ended questions. Table 1 includes the 95 participants who reported discrimination and racism-related comments. Participants ranged in age from 18 to 30 years (mean = 24.5 years), were primarily female (85.3%), and represented multiple racial/ethnic identities: 45.3% White, 34.7.% Asian, 7.4% Black, 4.2% Hispanic/Latinx, 6.3% mixed race, and 2.1% of another.

**Table 1:** Sample Characteristics (*n* = 95)

Characteristics	N Mean (SD) or %	
Age	95	24.5 (0.3)
Gender		
Female	81	85.3
Male	10	10.5
Non-Binary (includes Transsexual)	4	4.2
Race		
Asian or Asian American	33	34.7
Black or African American	7	7.4
Hispanic or Latinx	4	4.2
White, Caucasian, Anglo, European American	43	45.3
Multiracial	6	6.3
Other racial identity	2	2.1
Sexual Orientation		
Straight/Heterosexual	60	63.2
Gay, Lesbian	8	8.4
Bisexual	13	13.7
Asexual	4	4.2
Questioning	1	1.1
Self-Identify (pan sexual, queer), I prefer not to answer	9	9.5
Born in US		
Yes	76	80.0
No	19	20.0
First/Second Generation Immigrant		
Yes	38	41.3
No	54	58.7
Education Level		
High School graduate	1	1.1
Some college, vocational or technical school or associate college	29	30.5
College degree	33	34.7
Above college (Masters, some doctoral, doctoral)	32	33.7
Student Status		
Student	58	61.1
Non-student	37	38.9
Current Job Status		
Employed	61	64.2
Unemployed	34	35.8
Income Level		
No income	13	13.7
Under 25,000	46	48.4
25,000 - 49,999	16	16.8
50,000-74,999	13	13.7
75,000 - 99,999	1	1.05
100,000 -124,999	3	3.2
125,000 +	3	3.2
Married		
Yes	13	13.7
No	82	86.32
Housing Stability		
Not at all Stable	2	2.1
Slightly Stable	2	2.1
Moderately Stable	7	7.4
Stable	55	57.9
Extremely Stable	29	30.5

race (including American Indian/Native American participants). One in five participants was foreign-born (20.0%), 41.3% were first- or second-generation immigrants, 99% reported having at least attended some college, and 88.4% reported stable or extremely stable housing (88.4%).

Figure 1 describes the conceptual model of qualitative data analysis. We further defined the types of discrimination reported into three socioecological categories: societal, interpersonal, and intrapersonal. Affective reactions include fear, anxiety/distress, hopelessness/depression, and avoidance.

### Societal level (“political discrimination”)

Participants commented about witnessing COVID-19-related discrimination within social and political contexts through various platforms, including the news, newspapers, and social media. Participants made remarks pertaining to how U.S. political leaders have reinforced racial discrimination against the Chinese through the COVID-19 crisis. Comments also referred to the fractured current political system, contentious foreign policy, belligerent trade negotiations with China, and the anti-immigrant policy justifying the blame of China and A/AA for the cause and spread of COVID-19. This included the coining and repetition of culturally ethnocentric phrases such as “China Flu.”

### Interpersonal level (“direct” vs. “vicarious” discrimination)

We define the interpersonal level of discrimination as either direct or vicarious discrimination. First, direct discrimination has two manifestations: *overt* or blatant acts of prejudice (e.g., verbal or physical abuse, slurs, or threats); and *covert* or microaggressions, the unintentionally offensive acts of prejudice including subtle, derogatory slights, demeaning acts, or insults [24]. Second, vicarious discrimination was experienced both in-person and online. Participants reported being indirectly exposed to blatant acts of racism or discrimination targeted towards A/AA, such as witnessing or hearing about overt or covert incidents of direct discrimination. Based on our data, it appears that vicarious discrimination was witnessed by participants of all races, as both A/AA and non-Asian participants reported vicarious forms of discrimination.

### Intrapersonal level (“internalized discrimination”)

We classified internalized discrimination as discrimination at the intrapersonal level. Internalized racism was described as the acceptance of negative attitudes, beliefs, and stereotypes perpetuated by the society level and interpersonal level of discrimination [25].

Participant accounts of internalized racism frequently portrayed conflicting experiences of A/AA struggling with, accepting, and at times perpetuating anti-Asian stereotypes. A commonly reported example was the avoidance of A/AA businesses by those who were also A/AA who feared they might contract the virus through contact with other patrons. Participants reported feeling troubled that they were rejecting their own culture that they once embraced. This included feeling conflicted about their A/AA backgrounds.

Table 2 indicates the four different types of anti-Asian discrimination based on societal, interpersonal, and intrapersonal levels and the affective reactions from the discrimination reported by both A/AA and non-Asians. Out of 113 comments, 57.5% (*n* = 65) described the types of discrimination and 15.5% (*n* = 18) described an affective reaction to anti-Asian discrimination. A total of 5.3% (*n* = 6) comments were related to anti-Black discrimination (themes pertaining to non-Asian forms of discrimination were excluded given the focus of this analysis).

**Table 2:** Participants’ responses, constructs, and definitions

Types of Racism (*n* = 65)			
***Societal Level: Political discrimination***			
**Construct and Definition**	**Proportion of comments on types of racism** ***n*** **= 65**	**A/AA vs non-Asian**	**Selected Quotes**
**Political discrimination** Participant identifies a negative political climate in correlation with systemic racism and racist commentary made by political authorities.	*n* = 5 (7.7%)	**A/AA,*n*** **= 0, 0%** **non-Asian, *n* = 5, 7.7%**	**Reported by non-Asian (White)** I’d ask how Trump referring to COVID-19 as the “Wuhan Virus”, ‘Chinese Virus”, and “Kung Flu” impacts opinions. **Reported by non-Asian (White)** “The current social climate is very tumultuous with acts of racism from the police and political racism against Chinese people about the virus. I think it is important for these conversations to happen because being passive is just allowing these problems to continue to exist. People need to understand what is okay and what is not okay.”
***Interpersonal Level: Direct and Vicarious***			
**Construct and Definition**			**Selected Quotes**
**Direct:** **Overt Discrimination:** a blatant act of prejudice experienced by the participant directly (ie: verbal or physical abuse)	*n* = 1 (1.5%)	**A/AA, *n* = 1, 1.5%** **non-Asian, *n* = 0, 0.0%**	**Reported by A/AA:** “Got called a few racial slurs while walking in the streets because I am Chinese. They used the word “chink” quite a bit. Not sure if this is due to COVID or just generalized racism but I’m sure COVID didn’t help.”
**Direct:** **Covert microaggressions:** subtle, unintentionally offensive acts of prejudice experienced by the participant	*n* = 11 (16.9%)	**A/AA,*n*** **= 10, 15.4%** **non-Asian,*n*** **= 1, 1.5%**	**Reported by A/AA:** “I was at the mall with 3 other friends, we are all Asian. This one kiosk worker did not approach us about the product they were selling, but when a white couple walked by and they were offered the product. Being sick with something other than COVID and having to figure out how to avoid getting COVID while getting necessary care is stressful. It is also hard living with people who are not taking it seriously.”
**Vicarious** **In-person vicarious discrimination:** a blatant act of prejudice witnessed by the participant that was not personally experienced by the participant	*n* = 35 (53.8%)	**A/AA,*n*** **= 4, 6.2%** **non-Asian,*n*** **= 31, 47.7%**	**Reported by A/AA** **“**It was scary to hear about my acquaintance getting screamed at and physically assaulted (pushed) in the town where we grew up. I never experienced significant discrimination growing up so this seems so far out of the ordinary.” **Reported by non-Asian (Black):** “When I was on public transportation, I witnessed verbal abuse to someone I assumed was of Asian descent - an older male who was verbally assaulted by a young woman who was also a person of color. **Reported by non-Asian (White)** “I have a few younger cousins that are Asian American, as well as my partner who is Cuban/Cambodian, and they have all received extreme criticism for their races and have been verbally or physically assaulted because of their heritage. My youngest cousins are 2 and 5 years old and have been kicked out of their preschool because of their race.”
**Vicarious:** **Online vicarious discrimination:** participant recalls prejudicial comments made against Asian Americans on social media platforms	*n* = 9 (13.8%)	**A/AA, *n* = 4, 6.2%** **non-Asian, *n* = 5, 7.7%**	**Reported by A/AA (Total number 4)** “I think there’s a lot of xenophobia on social media where racism is rampant. While I understand social media users, as a whole, do not have a super high education attainment, but the ignorance and blunt racism still strike me.” **Reported by non-Asian (White)** “Admittedly, it has been frustrating to see and hear such incongruous “news” headlines and rhetoric surrounding this virus. There is an exorbitant amount of information that has been so clouded and distorted by political divide, international relations, and social commentary that I cannot form any resident theory about the ACTUAL state of things in the world, our country, my state, or my county. Such distortion and divide in place, the entire situation has only been made more stressful and contentious, which has made it personally more difficult to deal with this unfolding situation.”
***Intrapersonal Level: Internalized Discrimination***			
**Construct and Definition**			**Selected Quotes**
**Internalized discrimination:** People of colors’ internalized racism often leads to great conflict among and between them as other concepts of power—such as ethnicity, culture, nationality and class—are collapsed in misunderstanding	*n* = 4 (6.2%)	**A/AA,*n* = 4, 6.2%** **non-Asian, *n* = 0, 0%**	**Reported by A/AA (Total number 4):** “I personally would never call or support those who call COVID-19 the Chinese virus, but the source of the virus is important given where we live and how the world is built upon supremacy and racism. However, thinking about how my people might lose their loved ones because one of my fellow Asians decided to eat a bat or pangolin is uneasy to me and gives me a considerable amount of regret and stress. As a person who calls both countries home, I’m conflicted by the voices I hear every day. China is the place that gave me my childhood and my parents, yet it also allegedly spread the virus and concealed information from the world. On the other side is where I consider my home, and it’s currently being invaded.”
**Affective Reaction from Racism (*n* = 18)**			
**Fear**	*n* = 8 (44.4%)	**A/AA,*n* = 6, 33.3%** **non-Asian,*n* = 2, 11.1%**	**Reported by A/AA** “I haven’t faced discrimination, but I also haven’t left the house closed to for 4 weeks …. In fact, I fear leaving the house because I have heard reports of multiple attacks in and around my neighborhood.” **Reported by non-Asian (White)** “General fear and anger my Asian and Asian-American friends have that live in NYC, due to reports of assaults and experiencing verbal assault themselves.”
**Anxiety/Distress**	*n* = 5 (27.8%)	**A/AA,*n*** **= 2, 11.1%** **non-Asian,*n* = 3, 16.7%**	**Report from A/AA** “For me personally, I have a heightened sense of anxiety when I go out. I feel like I’m almost hyper-aware of non-Asians around me because I’m afraid someone is going to lash out at me because of my ethnicity. No one has verbally or physically attacked me yet but I’ve seen so many stories and videos of others being attacked. It scares me.” **Report from non-Asian (White)** My partner, who is Chinese, has experienced a higher level of anxiety, and I have experienced a higher level of anxiety because of their anxiety.
**Hopelessness/Depression**	*n* = 4 (22.2%)	**A/AA,*n* = 1, 5.6%** **non-Asians,*n* = 3, 16.7%**	**Report from A/AA** “Despite not being personally assaulted verbally/physically due to my race, those comments, videos or news articles online (both within China and outside China) made me feel depressed from time to time”. **Report from non-Asian** “It is exhausting and almost makes me feel hopeless. Even though racism is being exposed in institutions, public figures, and authority, there is denial and resistance to reform them which makes me feel distressed about it.”
**Avoidance**	*n* = 1 (5.6%)	**A/AA,*n* = 1, 5.6%** **non-Asians,*n* = 0, 0%**	**Report from A/AA (Total number 1)** “I have also adjusted my lifestyle (going out in public less or going with my white husband) to avoid discrimination. I stayed home from work one day because my asthma was acting up and I didn’t want to be seen coughing or showing any symptoms of illness because I am the only Asian staff member at my workplace of 100.”

Within the 113 open-ended responses, 49.6% of discrimination-related comments referenced interpersonal discrimination. Common excerpts included racial slurs and negative stereotypes towards the A/AA community in the form of jokes and direct slander. Within the discrimination-related comments, 11 comments (16.9%) were coded as covert microaggressions. Common themes within the microaggression responses included but were not limited to staring, avoidance by employees, and racial stereotyping. Numerous non-Chinese Asians also wrote that their nationality and race were questioned, despite not being Chinese and felt that the questioning was unnecessary and invasive. Those who did claim to be of Chinese descent perceived their identities to be negatively linked to the origin and spread of the virus by others.

Among the responses, vicarious discrimination (67.7%) was more commonly reported than direct discrimination. Both A/AA (*n* = 8, 12.3%) and non-Asians (*n* = 36, 55.4%) reported witnessing or hearing reports of anti-Asian discrimination such as physical attacks, verbal attacks, or harassment against friends, family, and strangers who are or are assumed to be Chinese. In particular, participants noted a surge in online vicarious racial discrimination against A/AA, which was described as rampant, ignorant, blunt, politically divisive, and distorted.

### Affective reactions

Table 2 indicates that out of 113 comments, 15.9% were about affective reactions in relation to anti-Asian discrimination. Two of the most common reactions were: fear and anxiety/distress. Due to the widespread reports of physical and verbal assaults heard on the media and reported by friends and family, A/AA reported that they feared leaving home even for routine tasks such as grocery shopping in fear that they may encounter direct discrimination.

These affective reactions to anti-Asian discrimination were also reported by non-Asian young adults. Although these respondents reported that they were not the likely target of anti-Asian racism or discrimination, their vicarious experiences resulted in a negative mental toll despite their non-Asian status. Non-Asian participants reported being afraid for their spouses, friends, or extended family members who are A/AA due to increasing anti-Asian racism. They also reported strong affective reactions both for themselves and for those in their close family or social circles. The comments, heavily influenced by highly publicized political racism, were shocking, and many participants reported feeling hopeless and exhausted about the inevitability of future encounters and frustrated by the lack of intervention towards the tense racial climate.

## Discussion

The purpose of this study was to explore racism and discrimination narratives written by A/AA and non-Asian U.S. young adults who participated in CARES [20] during the first six months of the COVID-19 pandemic in the U.S. To our knowledge, the data reported in this paper represent the first study to analyze open-ended survey comments pertaining to anti-Asian discrimination during the COVID-19 pandemic. Among the comments received by 611 participants, 95 A/AA and non-Asian individuals provided comments on COVID-19-related anti-Asian discrimination/racism. Such data on research questionnaires provide a valuable opportunity to further understand the pressing issues among the participants, yet are often neglected as a data source [26].

The biggest contribution of our study is that we examined both A/AA and non-Asian’s narratives regarding anti-Asian discrimination in the context of the COVID-19 pandemic. Our results show that both A/AA and non-Asian participants reported witnessing or hearing reports of anti-Asian discrimination. Moreover, both groups reported experiencing negative affective reactions to anti-Asian discrimination. Thus, anti-Chinese discrimination in the face of the COVID-19 pandemic may be more widespread and may have a greater negative societal impact than initially thought by affecting Chinese, non-Chinese Asian, and non-Asian populations in the U.S.

The political discrimination construct was mentioned only by non-Asians, not by any A/AA. On one hand, this may reflect the newfound or growing realization of anti-Asian discrimination among non-Asian young adults who may be more aware and vocal about racial justice. Another reason may be due to the features of our survey. The first open ended question (“Are there experiences we missed in the survey so far that you wish to describe?”) subsequently was asked after a series of COVID-19-related anti-Asian American discrimination questions, including whether they or their family members know people who have referred to the virus as the “Chinese/Wuhan virus,” whether they or their family were discriminated against because of their race/ethnicity, and if they heard someone making a comment about Chinese/Asian people being the source of the virus, for example [12]. While all were asked to respond to these survey questions (not just A/AA participants), it is possible that non-Asians felt the need to elaborate on their experiences given the emphasis of these particular items in the survey, whereas the items may have sufficiently captured the experiences among our A/AA participants.

The thematic analysis of text comments focusing on racism/discrimination allowed the emergence of two themes: types and affective reactions of discrimination. This study demonstrated that these multifaceted discrimination experiences are quite salient given the social climate in the U.S. The intensity of interpersonal discrimination was widespread, evidenced by interpersonal discrimination reported in a variety of locations, including on the street, on social media, in shopping malls, at school, and at work.

One of the most troubling aspects commented by participants was that anti-Asian discrimination was justified by institutional authority, referring to COVID-19 as “Wuhan Virus,” “China Virus,” and “Kung Flu.” The closest modern analogue to the types of racism, fear, and sense of insecurity, as well as the pervasiveness and regular occurrence of anti-Asian discrimination related to COVID-19 may be the ethnocentric experiences reported by Arab and Muslim Americans post-9/11, 2001 [27]. The discrimination that Arabs and Muslims experience range from name-calling to workplace discrimination, physical violence, and murder. This kind of pervasive ethnocentric response to world events can have detrimental impacts on individuals and communities. Both A/AA and non-Asians expressed their concerns that this normalization of racism against A/AA can lead to increased racial violence and perpetualization of anti-Asian racism.

Of particular note was the generalization of discrimination to non-Chinese Asian people who were misidentified as Chinese. This is unsurprising as historically, the discernment between specific origins in Asia during acts of discrimination or violence in the U.S. has been lacking. This is exemplified in the case of Vincent Chin who was murdered in 1982. Because of the growing Japanese auto industry with car imports in Detroit, Japanese people were scapegoated by Americans for their layoffs. Wrongly assuming he was Japanese (he was actually Chinese), a white mob approached Chin and beat him, where he later died from his injuries [28]. Similarly, A/AA as a whole group are being scapegoated today for the cause of the COVID-19 virus. Thus, Asians of various origins are often seen or treated as a single population despite their diversity [29, 30], which is evident in how A/AA people across national origins (such as Japanese, Korean, Chinese) report experiencing COVID-19-related discrimination events [31]. In the present study, non-Chinese Asians reported experiencing blatant racism and felt that it was unfair that all East Asians were treated as Chinese and became victims of racism. This may lead to resentment among the myriad of A/AA communities in the U.S., leaving the broader Asian minority group unprotected, isolated, marginalized, and further divided among themselves.

The description of reactions to anti-Asian discrimination during the COVID-19 pandemic should raise alarm. The most common affective reaction to racial discrimination was intensified fear for their own lives or loved ones. This fear was beyond a degree of minor discomfort; it immobilized people from going outside their own homes. This degree of hypervigilance due to racist experiences may have detrimental impacts on the mental health of people who experience it [32, 33].

Though the context of the COVID-19 pandemic has arguably intensified anti-Asian discrimination, it is foundational to the persistence of anti-Asian discrimination in the U.S. Asian Americans have been subject to various forms of social repression throughout U.S. history, including the codification of anti-Asian racism in U.S. law in the Chinese Exclusion Act of 1882, the Immigration Act of 1924, Japanese internment camps during World War II, and the ever-present treatment of Asian Americans as “perpetual foreigners” resulting in the sense of feeling like “aliens in their own lands” [29]. The perpetuation of Asian-specific stereotypes indicates that Asians are often not accepted as true Americans regardless of where they were born or how many years they have lived in the U.S. [34, 35]. These hostile social and structural environments are already challenging and create chronic stress, chronic illness, and psychological distress among A/AA [33]. Our study provides evidence that the COVID-19 pandemic substantiated an intense explosion of various types of discrimination and the harmful emotional tolls of anti-Asian discrimination to both A/AA and non-Asians. Combating discrimination against A/AA will take enormous and ongoing efforts: collaboration/coalition between multiple systems (educational systems, law enforcement, community organizations, and media campaigns) to dismantle harmful biases against A/AA, encourage and empower the A/AA community to build solidarity within the community, and establish solid allyships with Whites and other people of color. Thus, the elimination of anti-Asian discrimination will benefit those beyond the A/AA population, and we as a society must strive to achieve this goal.

### Limitations

The study’s limitations should be noted in the interpretation of the present study. Participants in response to open-ended questions volunteered experiences, however, such questions were not designed to elucidate detailed accounts. Future studies should employ more in-depth qualitative methods such as qualitative interviews to collect in-depth accounts of the social processes described in this analysis, including potential coping mechanisms through which people address the harmful effects of anti-Asian discrimination. In addition, this study focused on young adults ages 18–30. Therefore, like other qualitative studies, the results of this study are not generalizable to different age groups of individuals.

## Conclusion

Overall, these findings suggest that anti-Asian discrimination in the face of the COVID-19 pandemic is salient and impactful in both A/AA and non-Asian peoples’ lives. The perpetuation of racism and ethnocentrism by national leaders and community members tap into pre-existing anti-Asian sentiment and pose detrimental psychological impacts on both A/AA and non-Asian people. Additional research is needed to understand the social processes involved in these event-driven increases in racism, the impact of racism on health, and the design of interventions for reducing their incidence and preventing their detrimental psychological impact.

## Supplementary Information

**Additional file 1.**

## Data Availability

The datasets used and/or analyzed during the current study is available from the corresponding author upon reasonable request.

## Abbreviations

A/AA: Asian and Asian American
CARES: COVID-19 Adult Resilience Experiences Study
COVID-19: Coronavirus Disease of 2019
WHO: World Health Organization
IRB: Institutional Review Board
HCH: Hyeouk Chris Hahm
KG: Kana Garcia
AC: Anna Cavallino
