# Supplementary material for: Experiences of COVID-19-related anti-Asian discrimination and affective reactions in a multiple race sample of U.S. young adults
Source: BMC Public Health. 2021 Aug 18;21:1563. doi: 10.1186/s12889-021-11559-1 (PMC8371291; doi:10.1186/s12889-021-11559-1)
Supplement: Supplementary file 1 — Additional file 1. [file 12889_2021_11559_MOESM1_ESM.pdf]

CARES Wave 1 Survey

Start of Block: Screener

D1 Thank you for your interest in the COVID19 Adult Resilience Experiences Study (CARES)! This longitudinal study is a collaboration between the Boston University School of Social Work and Brigham and Women's Hospital, Harvard Medical School.   In this study, we will ask young adults about their personal experiences, physical health, and mental well being following the COVID-19 pandemic. This study will consist of a **30-40 minute online survey**. You may also be asked to participate in a 30-minute survey in July and October 2020. Some participants will also be asked to participate in a 20-minute video interview at each time point.
In order to determine your eligibility for our study, we ask that you share the following information about yourself. If you are eligible, you will be invited to proceed with our survey.

X→

elig_US (1) Are you living **in the United States**?

- ○ No (0)
- ○ Yes (1)

X→

elig_school (2) Are you currently receiving education from a **U.S. institution**?

- ○ No (0)
- ○ Yes (1)

X→

elig_age (3) Are you between the ages of 18-30?

- No (0)
- Yes (1)

End of Block: Screener

Start of Block: Ineligible

D4 Thank you for your interest in our research study! Unfortunately, based on your answers to our screening questions, we have determined that you are ineligible to participate.

End of Block: Ineligible

Start of Block: Consent

D5 Thank you for your interest in our research study, the COVID-19 Adult Resilience Experiences Study (CARES).

You are eligible to participate in this research study.

Below, please read our consent form for important information about your rights as a research participant. **At the end of this document, you will be asked to select "agree" if you agree to participate.**

C1 **CONSENT FORM**

Study Summary
The purpose of this research study is to learn about young adults' experiences, mental well being, and health outcomes following the COVID-19 pandemic.

Participants who take part in this research study will be in this research study for 6 months (April – October). During this time, participants may participate in the research study for up to three time points. These surveys and interviews will ask about stress, social support, and your experiences during the COVID-19 pandemic.

Participants taking part in this study will complete the following: A 30-40 minute online survey on Qualtrics about stress, social support, and your experiences during the COVID-19 pandemic. You may also be asked to participate in surveys at two additional time points in July/August and October/November.

At each time point, some participants may be asked to participate in an in-depth video interview online via Zoom (a video-conferencing application) following completion of their survey.

The risks of taking part in this research study are minimal, including possible discomfort when being asked to recall experiences of COVID-19 and potential loss of confidentiality. We make efforts to address these risks, as detailed in depth next page.

C2 **Introduction**

Please read this form carefully. The purpose of this form is to provide you with important information about taking part in a research study. If you have any questions about the research or any portion of this form, please ask us. Taking part in this research study is up to you. If you decide to take part in this research study, we will ask you to select "I agree" at the end of this form. We will provide a copy of the consent form as a PDF for download.

The two people in charge of this study are **Hyeouk Chris Hahm, PhD, LCSW** and **Cindy H. Liu, PhD**. Dr. Hahm can be reached at hahm@bu.edu or (617) 353-3925, and Dr. Liu may be reached at chliu@bwh.harvard.edu or (617) 525-4131. We will refer to these two people as the “researcher” throughout this form.

What should I know about this research study?
Participation in research is voluntary, which means that it is something for which you volunteer. It is your choice to participate in the study, or not to participate. If you choose to participate now, you may change your mind and stop participating later. If you decide not to participate, that decision will not result in any penalty or loss of benefits to which you are otherwise entitled.

Why is this study being done?
The purpose of this study is to learn about the effects of the COVID-19 pandemic on stress, resilience, and physical/mental health among young adults.

We are asking you to take part in this study because you are: Between the ages of 18-30 years old; Living within the United States and/or receiving education within the United States.
About **1000 participants** will take part in this research study nationwide.

Who is Funding the Study?
This study is not currently funded.

C3 **How long will I take part in this research study?**
We expect that you will be in this research study for approximately **6 months** for up to **three**

time points. At each time point, we may ask you to participate in a **30-45 minute online survey**. A select number of participants at each time point (20 participants) will be asked to participate in an additional 20-minute in-depth interview.

What will happen if I take part in this research study?
If you agree to take part in this study, we will ask you to sign this consent form before we conduct any study procedures. Below, please see the details for each time point. We will ask you to complete a 30-40 minute online Qualtrics survey regarding demographics, stress, social support, and experiences following the COVID-19 pandemic. A link to these surveys will be emailed to you prior to each time point. You may be contacted for two subsequent time points in July/August and October/November to additional online surveys. At each time point, we will select 20 participants to participate in a 20-minute online interview over Zoom, a video-conferencing application. During this interview, we will ask you about your personal experiences during the COVID-19 pandemic. You will have the option to conduct a video interview or audio only.
Are you willing to potentially be contacted for an interview?

○ Yes (please provide initials below): (4) ____

○ No (please provide initials below): (5) ____

C4 **What are the risks of taking part in this research study?**

Risks of Completing Tasks
You may get tired during the tasks. You can rest at any time.

Questionnaire/Survey Risks
You may feel emotional or upset when answering some of the questions. Tell the interviewer at any time if you want to take a break or stop the interview.

You may be uncomfortable with some of the questions and topics we will ask about. You do not have to answer any questions that make you feel uncomfortable.

Loss of Confidentiality
The main risk of allowing us to use and store your information for research is a potential loss of privacy. We will protect your privacy by labeling your information with a code and keeping the key to the code in a password-protected computer.

Reporting Suicidal Risk: If, during your participation of this study, we have reason to believe that you are at risk for being suicidal or otherwise harming yourself, we are required to take the

necessary actions. This may include notifying your doctor, your therapist, or other individuals. If this were to occur, we would not able to assure confidentiality.

You will be informed of any significant new findings developed during the course of this research which may affect your willingness to continue participation.

Are there any benefits from being in this research study?
There are no direct benefits to you from taking part in this research. However, others may benefit in the future from the information that is learned in this study.

What alternatives are available?
You may choose not to take part in this research study.

Study Participation and Early Withdrawal
Taking part in this study is your choice. You are free not to take part or to withdraw at any time for any reason. No matter what you decide, there will be no penalty or loss of benefit to which you are entitled. If you decide to withdraw from this study, the information that you have already provided will be kept confidential.

You may choose not to be in the study or to stop being in the study before it is over at any time. If you are a student at Boston University, your decision to participate or stop the study will not affect your class standing or your grades. You will not be offered or receive any special consideration if you take part in this research study.

Also, the researcher may take you out of this study without your permission. This may happen because: The researcher thinks it is in your best interest You can't make the required study visits Other administrative reasons

C5 **Audio/Video Recording**
We may ask you to participate in a video or audio-only interview (depending on your preference). If you are audio/video recorded it will be possible to identify you. We will store these recordings on our computer and only approved study staff will have access to the recordings. We will label these recordings with a code instead of your name. The key to the code connects your name to your recording. The researcher will keep the key to the code in a password-protected computer. The recordings will be kept until all data processing and analysis (interview transcriptions and subsequent analyses) are complete and will be stored for no more than one year after the analyses are completed.

Do you agree to allow us to audio/video record you during this study if you are willing to participate in an interview?

○ Yes (Please provide your initials below): (1) ____

○ No (Please provide your initials below): (2) ____

C6 **Note:** If we invite you to participate in an audio interview, we will also remind you of audio/video recording, when the interview takes place. You are allowed to change your mind at any time.

C7 **Use of Your Study Information**
Samples and private information collected from you during this study will NOT be used for future research studies or shared with other researchers for future research, even if the information identifying you are removed from the sample and/or private information.

How Will You Keep My Study Records Confidential?
We will keep the records of this study confidential by de-identifying your data to the extent possible. The identifiable information we do keep, such as your name and contact information when you sign the consent form, will be kept separate from our de-identified data. All data will be accessible to research staff only and will not be shared with anyone outside of the research group. We will make every effort to keep your records confidential. However, there are times when federal or state law requires the disclosure of your records.

The following people or groups may review your study records for purposes such as quality control or safety: The Researcher and any member of their research team The Institutional Review Board at Boston University. The Institutional Review Board is a group of people who review human research studies for safety and protection of people who take part in

the studies. The sponsor or funding agency for this study Federal and state agencies that oversee or review research Central University Offices
Data will be shared between Boston University and Brigham and Women's Hospital via Partners DropBox for Business, HIPAA compliant cloud-based storage. Qualtrics survey data will be accessible to research staff at both institutions and accessible only to members of the research team. All identifiable data and de-identified data for this research study will be stored separately on Partners DropBox for Business.

The results of this research study may be published or used for teaching. We will not include identifiable information on data that are used for these purposes.

C8 **Future Contact**
We may want to contact you in the future to see if you are interested in other studies taking place at Boston University.

May we contact you in the future?

○ Yes (Please provide your initials below): (1) ____

○ No (Please provide your initials below): (2) ____

C9 **Will I get paid for taking part in this research study?** We will compensate **one out of every 10** participants with a **$25 gift card** for completion of **each** 30-40 minute online survey. If you are selected to participate in the online Zoom interviews, you will be compensated with a **$20 gift card**.
What will it cost me to take part in this research study?
There are no costs to you for taking part in this research study.

Who do I ask if I have questions or concerns about this research study?
Please contact us with any concerns or questions about the research, or any research-related problems: Research Team: cares@bu.edu **Hyeouk Chris Hahm, PhD, LCSW** (Principal Investigator, Boston University School of Social Work) can be reached at hahm@bu.edu or (617) 353-3925. **Cindy Liu, PhD** (Co-Principal Investigator, Brigham and Women's Hospital, Harvard Medical School) may be reached at chliu@bwh.harvard.edu or (617) 525-4131. Study staff will be available from Monday-Friday, 9 am-5 pm EST.
If you have questions about your rights as a research participant, or if you have any complaints or concerns and want to speak with someone independent of the research team, you may contact the Boston University Charles River Campus IRB at 617-358-6115. The IRB Office

webpage has information where you can learn more about being a participant in research, and you can also complete a Participant Feedback Survey.

C10 **Statement of Consent**

I have read the information in this consent form including risks and possible benefits. I have been given the chance to ask questions. My questions have been answered to my satisfaction, and I agree to participate in the study.

○ I agree to participate in the study. (1)

○ I do not agree to participate in the study. (2)

End of Block: Consent

Start of Block: Denied participation

D5 Thank you for your time! We hope you stay safe and healthy during this time. Should you have any questions or concerns, please contact us at cares@bu.edu.

End of Block: Denied participation

Start of Block: Count 0

*

count0_1 Please complete the task below to ensure you are not a bot!

This is a number counting task. Please count the number of 0s shown in the picture below. This survey will terminate after three attempts.

This is your first attempt.

____

Skip To: CAPTCHA1 If Condition: Please complete the task be... Is Equal to 9. Skip To: Before you proceed to the survey, ple....

Page Break

count0_2 This is a number counting task. Please count the number of 0s shown in the picture below. This survey will terminate after three attempts.

This is your second attempt.

____

Skip To: CAPTCHA1 If Condition: This is a number counting t... Is Equal to 9. Skip To: Before you proceed to the survey, ple....

Page Break

JS *

count0_3 This is a number counting task. Please count the number of 0s shown in the picture below. This survey will terminate after three attempts.

This is your final attempt.

____

Skip To: End of Survey If Condition: This is a number counting t... Is Not Equal to 9. Skip To: End of Survey.

Page Break

JS

CAPTCHA1 Before you proceed to the survey, please complete the CAPTCHA below.

End of Block: Count 0

Start of Block: Count 1

*

count1_1
Please complete the task below to ensure you are not a bot!
This is a number counting task. Please accurately count the number of 1s shown in the picture below. This survey will terminate after three attempts.

This is your first attempt.

____

Skip To: CAPTCHA2 If Condition: Please complete the task be... Is Equal to 16. Skip To: Before you proceed to the survey, ple....

Page Break

JS *

count1_2 This is a number counting task. Please count the number of 1s shown in the picture below. This survey will terminate after three attempts.

This is your <u>second</u> attempt.

____

Skip To: CAPTCHA2 If Condition: This is a number counting t... Is Equal to 16. Skip To: Before you proceed to the survey, ple....

Page Break

count1_3 This is a number counting task. Please count the number of 1s shown in the picture below. This survey will terminate after three attempts.

This is your final attempt.

____

Skip To: End of Survey If Condition: This is a number counting t... Is Not Equal to 16. Skip To: End of Survey.

Page Break

JS

CAPTCHA2 Before you proceed to the survey, please complete the CAPTCHA below.

End of Block: Count 1

Start of Block: Demographics

ID Thank you for agreeing to participate in CARES (COVID-19 Adult Resilience Experience Study)! This is the **first of three online surveys** you will complete in the next 6 months. The following questions will ask about your demographics, relocation experiences, stress, social support, and health. This survey may take some time to complete - we estimate that it may take around 30-40 minutes of your time.

One out of every 10 participants who complete the survey will receive a $25 gift card to Amazon.

At the end of this survey, we will ask you to provide your **e-mail address** in order for us to contact you in regard to a) study follow-ups in July and October, and b) about the gift card raffle. Providing your email address is optional.

D1 **Demographics**

JS * X→

DOB **What is your date of birth?**

	Month	Day	Year
Please Select: (DOB)	▼ January (1 ... December (12)	▼ 1 (1 ... 31 (31)	▼ 1900 (1 ... 2049 (150)

gender **What is your gender?**

- ○ Woman (1)
- ○ Man (2)
- ○ Trans woman (3)
- ○ Trans man (4)
- ○ Self-identify (please indicate): (5) ____

---

sexorient **What is your sexual orientation?**

- ○ Asexual (1)
- ○ Bisexual (2)
- ○ Gay (3)
- ○ Lesbian (4)
- ○ Questioning (5)
- ○ Straight/Heterosexual (6)
- ○ Self-identify (please indicate): (7) ____
- ○ I prefer not to answer (8)

---

hispanic **Are you of Hispanic origin?**

- ○ No (0)
- ○ Yes (1)

race **What is your race/ethnicity?**

- ◯ Asian or Asian American, including Chinese, Japanese, and others (1)
- ◯ Black or African American (2)
- ◯ Hispanic or Latinx, including Mexican American, Central American, and others (3)
- ◯ White, Caucasian, Anglo, European American; not Hispanic (4)
- ◯ American Indian/Native American (5)
- ◯ Mixed; Parents are from two different groups (6)
- ◯ Other (please indicate): (7) ____

X→

parentrace **My current parents/guardians' ethnicities are:**

	Asian or Asian American, including Chinese, Japanese, and others (1)	Black or African American (2)	Hispanic or Latinx, including Mexican American, Central American, and others (3)	White, Caucasian, Anglo, European American; not Hispanic (4)	American Indian/Native American (5)	Mixed; Parents are from two different groups (6)	Other (7)
Parent 1 (1)	◯	◯	◯	◯	◯	◯	◯
Parent 2 (2)	◯	◯	◯	◯	◯	◯	◯

Display This Question:

If My current parents/guardians' ethnicities are: = Parent 1 [Other]

parent1_0 **If selected "Other" for Parent 1, please indicate:**

____

Display This Question:

If My current parents/guardians' ethnicities are: = Parent 2 [Other]

parent2_0 **If selected "Other" for Parent 2, please indicate:**

____

X→

bioparent_c **Are your current parent/guardians your biological parents?**

○ No (0)

○ Yes (1)

Display This Question:

If Are your current parent/guardians your biological parents? = No

X→

bioparent_race **What are your biological parent's ethnicities:**

	Asian or Asian American, including Chinese, Japanese, and others (1)	Black or African American (2)	Hispanic or Latinx, including Mexican American, Central American, and others (3)	White, Caucasian, Anglo, European American; not Hispanic (4)	American Indian/Native American (5)	Mixed; Parents are from two different groups (6)	Other (7)
Parent 1 (1)	○	○	○	○	○	○	○
Parent 2 (2)	○	○	○	○	○	○	○

Display This Question:

If What are your biological parent's ethnicities: = Parent 1 [Other]

biopar1 **If selected "Other" for Parent 1, please indicate:**

____

Display This Question:

If What are your biological parent's ethnicities: = Parent 2 [Other]

biopar2 **If selected "Other" for Parent 2, please indicate:**

____

residence **What do you consider as your primary residence?**

- ○ Within the U.S. (1)
- ○ Outside the U.S. (2)

Display This Question:

If What do you consider as your primary residence? = Within the U.S.

USres **Please indicate the <u>U.S. state</u> (two-letter state abbreviation) of your primary residence:**

____

Display This Question:

If What do you consider as your primary residence? = Outside the U.S.

nonUSres **Please indicate the <u>country</u> of your primary residence:**

____

usborn **Were you born within the U.S.?**

- ◯ No (0)
- ◯ Yes (1)

Display This Question:

If Were you born within the U.S.? = No

nonUSage **How old were you when you first arrived the U.S.?**

- ◯ Under 12 (1)
- ◯ 12 and over (2)

Display This Question:

If How old were you when you first arrived the U.S.? , 12 and over Is Displayed

And How old were you when you first arrived the U.S.? , Under 12 Is Displayed

homecountry **Please indicate your home country:**

____

---

immigrant **Do you consider yourself to be a first or second generation immigrant (you or one of your parents immigrated to the U.S. from a different country)?**

- ◯ No (0)
- ◯ Yes (1)

---

married **Are you married?**

◯ No (0)

◯ Yes (1)

---

children **Do you currently have children?**

◯ No (0)

◯ Yes (1)

Display This Question:

If Do you currently have children? = Yes

child_num **How many children?**

____

---

income **Last year, what was your <u>individual</u> income before taxes in USD? Please include income from all sources such as social security/disability, stocks, alimony and child support in the past year.**

- ○ No income (0)
- ○ Under $25,000 (1)
- ○ $25,000-50,000 (2)
- ○ $50,000-74,999 (3)
- ○ $75,000-99,999 (4)
- ○ $100,000-124,999 (5)
- ○ $125,000-149,999 (6)
- ○ $150,000-174,999 (7)
- ○ $175,000-199,999 (8)
- ○ $200,000-224,999 (9)
- ○ $225,000-249,999 (10)
- ○ $250,000+ (11)

---

famSSS **Next, think of this ladder as representing where people stand in the country from which you were raised.**

At the **top** of the scale are the people who are the best off - those who have the most money, the most education, and the most respected jobs. At the **bottom** of the scale are the people who are the worst off - who have the least money, the least education, and the least respected jobs or no job. The higher up you are on this ladder, the closer you are to the people at the very top; the lower you are, the closer you are to the people at the very bottom.

Where would you place <u>your family when you were growing up</u> on this ladder?

▼ 1 (1) ... 10 (10)

---

prevevict **Have you ever been forced to relocate or evicted?**

- ○ No (0)
- ○ Yes (1)

---

housing **How stable is your current housing?**

- ○ Not at all stable (1)
- ○ Slightly stable (2)
- ○ Moderately stable (3)
- ○ Stable (4)
- ○ Extremely stable (5)

---

insurance **During this past year, did you have health insurance?**

- No (0)
- Yes, part of the time (1)
- Yes, all the time (2)

---

insur_curr **Do you currently have health insurance?**

- No (0)
- Yes (1)
- I don't know (2)

---

insur_source **This past year, what was your primary source of health insurance?**

- I did not have health insurance (0)
- Private insurance through my school (e.g., student health insurance) (1)
- Private insurance from my job or the job of my spouse (2)
- Private insurance from my parents (3)
- Private health insurance from the Health Insurance Marketplace or Healthcare.gov (4)
- Medicaid (5)
- State-specific option (other government plan or program such as SCHIP/CHIP) (6)
- State-specific option (other government plan or program not listed above) (7)
- State-specific option (TRICARE or other military health care) (8)
- State-specific option (HIS or tribal) (9)
- Other health insurance, please indicate: (10) ____

---

credit **Financial well being may affect health. How would you rate your consumer credit?**

- Poor (1)
- Fair (2)
- Good (3)
- Very good (4)
- Excellent (5)
- Do not know/do not have any consumer credit (6)
- Prefer not to respond (7)

welfare **In your household, there was at least one time when the household:**

	As a child (up to age 11)			As a teenager (age 12-18)			As an adult (age 19 to present)		
	Yes (1)	No (0)	Don't know (2)	Yes (1)	No (0)	Don't know (2)	Yes (1)	No (0)	Don't know (2)
Did not have enough money for food or housing (welfare_1)	○	○	○	○	○	○	○	○	○
Received public assistance or welfare (welfare_2)	○	○	○	○	○	○	○	○	○

mhdiag **Have you ever been diagnosed with the following? Please scroll to the right to see all response options.**

	No (0)	Suspected, but not diagnosed (1)	Yes, diagnosed but not treated (2)	Yes, diagnosed and treated (3)
ADHD (1)	○	○	○	○
Generalized Anxiety Disorder (2)	○	○	○	○
Depression (3)	○	○	○	○
Insomnia (4)	○	○	○	○
Obsessive Compulsive Disorder (OCD) (5)	○	○	○	○
Panic Disorder (6)	○	○	○	○
Post-traumatic Stress Disorder (PTSD) (7)	○	○	○	○
Substance abuse or addiction (alcohol or other drugs) (8)	○	○	○	○
Other mental health condition, please indicate: (9)	○	○	○	○

education **What is your highest level of education?**

- Completed part of high school but didn't finish (1)
- GED (2)
- High school graduate (3)
- Some college, vocational or technical school (4)
- Vocational or technical school graduate (5)
- Associate's degree (6)
- College degree (BS/BA) (7)
- Some advanced work, but no graduate degree (8)
- Master's degree (MS/MA) (9)
- Some work toward doctorate or advanced degree (10)
- Doctoral or advanced degree (i.e., MD, JD, DO, DDS, or PhD) (11)

---

student **Are you currently a student?**

- No (0)
- Yes (1)

Display This Question:

If Are you currently a student? = Yes

stud_type **I am currently a...**

- ◯ College student (undergraduate) (1)
- ◯ Graduate student (2)

Display This Question:

If I am currently a... = College student (undergraduate)

undergrad **What year are you?**

- ◯ 1st year undergraduate (1)
- ◯ 2nd year undergraduate (2)
- ◯ 3rd year undergraduate (3)
- ◯ 4th year undergraduate (4)
- ◯ 5th year undergraduate (5)
- ◯ Other, please indicate: (6) ____

Display This Question:

If I am currently a... = Graduate student

gradstud **What year are you?**

- 1st year graduate student (1)
- 2nd year graduate student (2)
- 3rd year graduate student (3)
- 4th year graduate student (4)
- 5th year graduate student (5)
- 6 or more years, graduate student (6)
- Other, please indicate: (7)

X→

jobstatus **What is your current job status?**

- Employed (1)
- Not employed (0)

Display This Question:

If What is your current job status? = Employed

employed **During the COVID-19 pandemic, I am currently...**

- Employed and working as I normally do (1)
- Employed but my job responsibilities have changed (e.g., working remotely) (2)
- Employed but cannot perform my job responsibilities due to the COVID-19 pandemic (3)

Display This Question:

If What is your current job status? = Employed

workhrs **How many hours do you work per week?**

- ◯ 40 or more hours per week (1)
- ◯ 21-39 hours per week (2)
- ◯ 20 hours per week or less (3)

Display This Question:

If What is your current job status? = Not employed

unemployed **During the COVID-19 pandemic...**

- ◯ I have been unemployed due to COVID-19 pandemic-related circumstances (e.g., economic downturn) (1)
- ◯ I am unemployed for other reasons (2)
- ◯ I am not employed an am **not** currently seeking a job (3)

Display This Question:

If Are you currently a student? = Yes

school **What school do you currently attend?**

- ◯ Name of Institution (1) ____
- ◯ City (2) ____
- ◯ State (3) ____

Display This Question:

If Are you currently a student? = Yes

schoolstat **What is your school enrollment status?**

- Full time (1)
- Part time (2)
- Other (3)

Display This Question:

If Are you currently a student? = Yes

intlstudent **Are you an international student?**

- No (0)
- Yes (1)

Display This Question:

If Are you currently a student? = Yes

studemploy **During the academic year, how many hours a week do you typically work for pay?**

- 0 hours (0)
- 1-9 hours (1)
- 10-19 hours (2)
- 20-29 hours (3)
- 30-39 hours (4)
- 40 hours (5)
- More than 40 hours (6)

Display This Question:

If Are you currently a student? = Yes

finaid **Do you currently receive financial aid?**

- No (0)
- Yes (1)

Display This Question:

If Do you currently receive financial aid? = Yes

finaid_need **Is this financial aid need-based (not merit-based)?**

- No (0)
- Yes (1)

Display This Question:

If Is this financial aid need-based (not merit-based)? , Yes Is Displayed

finaid_per **Please estimate the percentage of your tuition covered by financial aid.**

Please indicate %:

____

Display This Question:

If I am currently a... = College student (undergraduate)

firstgen **Are you a first-generation college student (i.e., your parents have not completed a bachelor's degree)?**

- No (0)
- Yes (1)

Display This Question:

If I am currently a... = College student (undergraduate)

transfer **Have you transferred to this college or university in the *last 12 months*?**

- No (0)
- Yes (1)

Display This Question:

If Have you transferred to this college or university in the last 12 months? = Yes

transfer_school **What was the previous school you attended?**

Please indicate:

- Name of Institution (1) ____
- City (2) ____
- State (3) ____

Display This Question:

If Are you currently a student? = Yes

acyrhousing **Where did you live for school during the most recent academic year (2019-2020)?**

◯ Campus residence hall (1)

◯ Fraternity or sorority house (2)

◯ Other college/university housing (3)

◯ Parent/guardian's home (4)

◯ Other off-campus housing (5)

◯ Other, please indicate: (6) ____

Display This Question:

If Are you currently a student? = Yes

relocation **Were you mandated to vacate from your residence by your university due to the COVID-19 outbreak and thus required to find new living arrangements?**

◯ No (0)

◯ Yes (1)

Display This Question:

If Were you mandated to vacate from your residence by your university due to the COVID-19 outbreak a... = Yes

reloc_days **How many days did you have to move out of your residence following your university's campus closing announcement?**

Please indicate:

____

Display This Question:

If Were you mandated to vacate from your residence by your university due to the COVID-19 outbreak a... = Yes

reloc_stress **How stressed were you about finding a place to stay following your university's campus closure?**

- ◯ Not stressed at all (1)
- ◯ A little stressed (2)
- ◯ Somewhat stressed (3)
- ◯ Stressed (4)
- ◯ Extremely stressed (5)

Page Break

Display This Question:

If Were you mandated to vacate from your residence by your university due to the COVID-19 outbreak a... = Yes

location
Now we are going to ask your current location. Where do you live right now?

	Living Space	Was payment required for you to stay at this location		Location	
		No (1)	Yes (2)	In the U.S. (1)	Outside the U.S. (2)
Current residence (currlocation)	▼ Parent/guardian's home (1 ... Other (10)	○	○	○	○

Display This Question:

If Now we are going to ask your current location. Where do you live right now? : Living Space = Current residence [Other]

location_oth **If you selected "other" for your __current__ living space, please indicate:**

____

Display This Question:

If Now we are going to ask your current location. Where do you live right now? : Location = Current residence [In the U.S.]

locationzip **Please provide the zip code of your __current__ residence.**

○ Zip code (1) ____

Display This Question:

If Now we are going to ask your current location. Where do you live right now? : Location = Current residence [Outside the U.S.]

loc_country **Please provide the country of your current location.**

○ Country (1) ____

Display This Question:

If Were you mandated to vacate from your residence by your university due to the COVID-19 outbreak a... = Yes

X→

reloc_multi **Is the current location the first place you moved to after the university announced that you had to leave campus?**

○ No (0)

○ Yes (1)

Display This Question:

If Is the current location the first place you moved to after the university announced that you had... = No

reloc_moves **How many other places did you stay at before your current location?**

▼ 1 (1) ... 5+ (5)

Display This Question:

If Is the current location the first place you moved to after the university announced that you had... = No

reloc_housing **For each place you stayed, indicate the number of days you stayed at each place, the type of housing, whether payment was required, and the location (in the U.S. or outside the U.S.)**

	Number of Days	Type of housing	Was payment required for you to stay at this location?	Location

	Please indicate: (1)		No (1)	Yes (2)	In the U.S. (1)	Outside the U.S. (2)
First residence (reloc_housing_1)		▼ Parent/guardian's home (1 ... Other (10)	◯	◯	◯	◯
Second residence (reloc_housing_2)		▼ Parent/guardian's home (1 ... Other (10)	◯	◯	◯	◯
Third residence (reloc_housing_3)		▼ Parent/guardian's home (1 ... Other (10)	◯	◯	◯	◯
Fourth residence (reloc_housing_4)		▼ Parent/guardian's home (1 ... Other (10)	◯	◯	◯	◯
Fifth residence (reloc_housing_5)		▼ Parent/guardian's home (1 ... Other (10)	◯	◯	◯	◯

Display This Question:

If For each place you stayed, indicate the number of days you stayed at each place, the type of hous... : Type of housing = First residence [Other]

reloc_hous1 **If you selected "other" for your <u>first</u> residence, please indicate:**

____

Display This Question:

If For each place you stayed, indicate the number of days you stayed at each place, the type of hous... : Location = First residence [In the U.S.]

reloc_hous1US **Please provide the location of your <u>first</u> residence.**

○ City (1) ____

○ State (3) ____

Display This Question:

If For each place you stayed, indicate the number of days you stayed at each place, the type of hous... : Location = First residence [Outside the U.S.]

reloc_hous1nonUS **Please provide the location of your <u>first</u> residence.**

○ Country (1) ____

Display This Question:

If For each place you stayed, indicate the number of days you stayed at each place, the type of hous... : Type of housing = Second residence [Other]

reloc_hous2 **If you selected "other" for your <u>second</u> residence, please indicate:**

____

Display This Question:

If For each place you stayed, indicate the number of days you stayed at each place, the type of hous... : Location = Second residence [In the U.S.]

reloc_hous2US **Please provide the zip code of your <u>second</u> residence.**

◯ Zip code (1) ____

Display This Question:

If For each place you stayed, indicate the number of days you stayed at each place, the type of hous... : Location = Second residence [Outside the U.S.]

reloc_hous2nonUS **Please provide the location of your <u>second</u> residence.**

◯ Country (1) ____

Display This Question:

If For each place you stayed, indicate the number of days you stayed at each place, the type of hous... : Type of housing = Third residence [Other]

reloc_hous3 **If you selected "other" for your third residence, please indicate:**

____

Display This Question:

If For each place you stayed, indicate the number of days you stayed at each place, the type of hous... : Location = Third residence [In the U.S.]

reloc_hous3US **Please provide the zip code of your <u>third</u> residence.**

◯ Zip code (1) ____

Display This Question:

If For each place you stayed, indicate the number of days you stayed at each place, the type of hous... : Location = Third residence [Outside the U.S.]

reloc_hous3nonUS **Please provide the location of your <u>third</u> residence.**

◯ Country (1) ____

Display This Question:

If For each place you stayed, indicate the number of days you stayed at each place, the type of hous... : Type of housing = Fourth residence [Other]

reloc_hous4 **If you selected "other" for your <u>fourth</u> residence, please indicate:**

____

Display This Question:

If For each place you stayed, indicate the number of days you stayed at each place, the type of hous... : Location = Fourth residence [In the U.S.]

reloc_hous4US **Please provide the zip code of your <u>fourth</u> residence.**

○ Zip code (1) ____

Display This Question:

If For each place you stayed, indicate the number of days you stayed at each place, the type of hous... : Location = Fourth residence [Outside the U.S.]

reloc_hous4nonUS **Please provide the location of your <u>fourth</u> residence.**

○ Country (1) ____

Display This Question:

If For each place you stayed, indicate the number of days you stayed at each place, the type of hous... : Type of housing = Fifth residence [Other]

reloc_hous5 **If you selected "other" for your <u>fifth</u> residence, please indicate:**

____

Display This Question:

If For each place you stayed, indicate the number of days you stayed at each place, the type of hous... : Location = Fifth residence [In the U.S.]

reloc_hous5US **Please provide the zip code of your fifth residence.**

○ Zip code (1) ____

Display This Question:

If For each place you stayed, indicate the number of days you stayed at each place, the type of hous... : Location = Fifth residence [Outside the U.S.]

reloc_hous5nonUS **Please provide the location of your fifth residence.**

○ Country (1) ____

Display This Question:

If Were you mandated to vacate from your residence by your university due to the COVID-19 outbreak a... = Yes

X→

reloc_items **After being told to relocate, did you have to leave behind any valuable personal belongings?**

○ No (0)

○ Yes (1)

Display This Question:

If After being told to relocate, did you have to leave behind any valuable personal belongings? = Yes

reloc_items2 **How stressed were you about gathering your personal belongings?**

○ Not stressed at all (1)

○ A little stressed (2)

○ Somewhat stressed (3)

○ Stressed (4)

○ Extremely stressed (5)

End of Block: Demographics

Start of Block: FSS

finance1_T1 We are interested in how you feel about the following statements. Read each statement carefully. Indicate how you feel about each statement.

	Strongly disagree (1)	Mildly disagree (2)	Mildly agree (3)	Strongly agree (4)
1. I feel stressed about my personal finances in general. (finance_T1_1)	o	o	o	o
2. I worry about being able to pay monthly expenses. (finance_T1_2)	o	o	o	o
3. I worry about having enough money to pay for school. (finance_T1_3)	o	o	o	o

finance2_T1 **How much stress do the following items cause you?**

	Does not apply/No debt (0)	None (1)	Small amount (2)	Medium amount (3)	Large amount (4)	Extreme amount (5)
4. The total amount of money you owe (finance_T1_4)	○	○	○	○	○	○
5. Credit card debt (finance_T1_5)	○	○	○	○	○	○
6. Student loan debt (finance_T1_6)	○	○	○	○	○	○

End of Block: FSS

Start of Block: COVID-19 Experiences

D2 **COVID-19 Experiences**

diagnosisT1 **Have you been diagnosed with COVID-19?**

○ No, I have not been diagnosed with COVID-19 (0)

○ Yes, I received a test and tested positive for COVID-19 (1)

○ Yes, I have been presumed to have COVID-19 (based on doctor's diagnosis) (2)

○ I am currently being tested for COVID-19 and am waiting for my results (3)

○ I do not know (4)

diag_othT1 **Has a significant person in your life (e.g., close friend/acquaintance of family member) been diagnosed with COVID-19?**

- ○ No (0)
- ○ Yes (tested positive or presumed to have COVID-19) (1)
- ○ I do not know (2)

COVIDriskT1 **How much do you agree with the following statements about your risk for contracting COVID-19?**

	Strongly disagree (1)	Disagree (2)	Somewhat agree/Somewhat disagree (3)	Agree (4)	Strongly agree (5)
a. I am highly likely to contract COVID-19 during this pandemic. (COVIDriskT1_1)	○	○	○	○	○
b. I get sick more easily than other people my age. (COVIDriskT1_2)	○	○	○	○	○
c. If I developed COVID-19, I would not be able to manage daily activities. (COVIDriskT1_3)	○	○	○	○	○
d. COVID-19 is a life-threatening disease for me. (COVIDriskT1_4)	○	○	○	○	○

COVIDworryT1 **How worried do you feel about the following statements?**

I am worried...

	Not worried at all (1)	A little worried (2)	Somewhat worried (3)	Worried (4)	Very worried (5)
a. ...that I won't have enough groceries during city lockdowns/social distancing protocols. (COVIDworryT1_1)	○	○	○	○	○
b. ...that I will not be able to obtain a COVID-19 test if I become sick. (COVIDworryT1_2)	○	○	○	○	○
c. ...that I will not be treated for COVID-19 if I contract it. (COVIDworryT1_3)	○	○	○	○	○
d. ...about keeping in touch with loved ones during social distancing protocols. (COVIDworryT1_4)	○	○	○	○	○
e. ...about maintaining employment during the subsequent economic downturn. (COVIDworryT1_5)	○	○	○	○	○
f. ...about having enough money to pay for rent and buy basic necessities. (COVIDworryT1_6)	○	○	○	○	○

COVIDgriefT1 **Please rate the extent to which you agree or disagree with the following statements about your COVID-19 experiences using the options below.**

	Strongly disagree (1)	Disagree (2)	Somewhat agree/Somewhat disagree (3)	Agree (4)	Strongly agree (5)
a. I will miss out on significant life events due to COVID-19. (1)	◯	◯	◯	◯	◯
b. I worry about losing touch with my friends due to social distancing. (2)	◯	◯	◯	◯	◯
c. I have lost vital resources due to COVID-19 (e.g., housing, mentorship, food access, health care). (3)	◯	◯	◯	◯	◯
d. I feel stunned or dazed over what happened. (4)	◯	◯	◯	◯	◯
e. I feel that life is empty. (5)	◯	◯	◯	◯	◯
f. I feel bitter that COVID-19 caused me to experience loss in my routines and activities. (6)	◯	◯	◯	◯	◯

COVIDprevT1 **How effective do you perceive the following actions as preventative measures for contagion?**

	Not at all effecitve (1)	A little effective (2)	Somewhat effective (3)	Effective (4)	Very effective (5)
a. Washing hands frequently (1)	○	○	○	○	○
b. Wearing a face mask and/or gloves (2)	○	○	○	○	○
c. Social distancing (i.e., switching to online classes, working from home, maintaining 6 feet away from others) (3)	○	○	○	○	○
d. Closing schools and non-essential businesses (4)	○	○	○	○	○
e. Social isolation of infected individuals (i.e., people who are infected are kept away from those who are not infected at home or a hospital/care facility) (5)	○	○	○	○	○

COVIDcommT1 **Please complete the following table regarding communication with family and friends following the COVID-19 pandemic.**

	How frequently do you communicate with them?	How does your frequency of communication compare to before COVID-19?
Family/Relatives (COVIDcommT1_1)	▼ Multiple times a day (1 ... Once a month (5)	▼ We communicate less (1 ... We communicate more (3)
Friends/Peers (COVIDcommT1_2)	▼ Multiple times a day (1 ... Once a month (5)	▼ We communicate less (1 ... We communicate more (3)

COVIDnameT1 **Do you personally know people (e.g., friends and family) who have referred to the virus as the "Chinese/Wuhan virus" etc.?**

- No (0)
- Yes (1)

chin_virT1 **Do you agree with using the term "Chinese/Wuhan virus" to describe the virus?**

- Strongly disagree (1)
- Disagree (2)
- Neither agree nor disagree (3)
- Agree (4)
- Strongly agree (5)

COVID_discrimT1 **Have you experienced discrimination that was specifically directed at you or your family due to your race/ethnicity because of COVID-19 in the following ways?**

	No (0)	Yes (1)
a. Someone has actively avoided physical contact with me or my family because of my or my family member's race/ethnicity (e.g., on the street, inviting me to their home). (2)	o	o
b. Someone has made a comment about Chinese/Asian people being the source of the virus. (3)	o	o
c. Someone has made a comment about Chinese/Asian people being dirty. (4)	o	o
d. Someone has made a comment that they avoid eating Chinese/Asian food because they are worried about contracting COVID-19. (5)	o	o
e. Someone has made a comment about Chinese/Asian eating habits (e.g., eating bat soup) (6)	o	o
f. I or my family member have been verbally assaulted because of my or my family member's race/ethnicity due to COVID-19. (7)	o	o
g. I or my family member have been physically assaulted because of my or my family member's race/ethnicity due to COVID-19. (8)	o	o
h. Other (please indicate): (9)	o	o

Display This Question:

If Have you experienced discrimination that was specifically directed at you or your family due to y... = Yes

discrim_upsetT1 **How upset were you by these incidents of discrimination?**

	Not at all (1)	A little upset (2)	Somewhat upset (3)	Upset (4)	Very upset (5)
Have you experienced discrimination that was specifically directed at you or your family due to y... = a. Someone has actively avoided physical contact with me or my family because of my or my family member's race/ethnicity (e.g., on the street, inviting me to their home). [Yes]					
a. Someone has actively avoided physical contact with me or my family because of my or my family member's race/ethnicity (e.g., on the street, inviting me to their home). (2)	○	○	○	○	○
Have you experienced discrimination that was specifically directed at you or your family due to y... = b. Someone has	○	○	○	○	○

made a comment about Chinese/Asian people being the source of the virus. [Yes]					
b. Someone has made a comment about Chinese/Asian people being the source of the virus. (3)					
Have you experienced discrimination that was specifically directed at you or your family due to y... = c. Someone has made a comment about Chinese/Asian people being dirty. [Yes]	○	○	○	○	○
c. Someone has made a comment directed at me or someone else about Chinese/Asian people being dirty. (4)					
Have you experienced discrimination that was specifically directed at you or your family due to y... = d. Someone has made a comment that they avoid	○	○	○	○	○

eating Chinese/Asian food because they are worried about contracting COVID-19. [Yes]					
d. Someone has made a comment that they avoid eating Chinese/Asian food because they are worried about contracting COVID-19. (5)					
Have you experienced discrimination that was specifically directed at you or your family due to y... = e. Someone has made a comment about Chinese/Asian eating habits (e.g., eating bat soup) [Yes]	○	○	○	○	○
e. Someone has made a comment about Chinese/Asian eating habits (e.g., eating bat soup) (6)					
Have you experienced discrimination that was specifically directed at you or your family due to y... = f. I	○	○	○	○	○

or my family member have been verbally assaulted because of my or my family member's race/ethnicity due to COVID-19. [Yes]					
f. I or my family member have been verbally assaulted because of my or my family member's race/ethnicity due to COVID-19. (7)					
Have you experienced discrimination that was specifically directed at you or your family due to y... = g. I or my family member have been physically assaulted because of my or my family member's race/ethnicity due to COVID-19. [Yes]	○	○	○	○	○
g. I or my family member have been physically assaulted because of my or my family member's race/ethnicity					

due to COVID-19. (8)					
Have you experienced discrimination that was specifically directed at you or your family due to y... = h. Other (please indicate): [Yes]	○	○	○	○	○
h. Other (please indicate): (9)					

End of Block: COVID-19 Experiences

Start of Block: Experience

comments **Are there experiences we missed in the survey so far that you wish to describe?**

____

____

____

____

____

End of Block: Experience

Start of Block: SF-12

D3 This survey asks for your views about your health. This information will help keep track of how you feel and how well you are able to do your usual activities. If you are unsure how to answer a question, please give the best answer you can.

health1_T1 **In general, would you say your health is:**

- Excellent (5)
- Very good (4)
- Good (3)
- Fair (2)
- Poor (1)

health2_T1 **Does your health now limit you in these activities?**

	NO, not limited at all (3)	YES, limited a little (2)	YES, limited a lot (1)
**Moderate activities** such as moving a table, pushing a vacuum cleaner, bowling, or playing golf. (1)	○	○	○
Climbing **several** flights of stairs. (2)	○	○	○

health3_T1
The following questions ask about your experiences during the **past 4 weeks**.
Have you had any of the following problems with your work or other regular daily activities...

As a result of your physical health?

	NO (2)	YES (1)
**Accomplished less** than you would like. (1)	○	○
Were limited in the **kind** of work or other activities. (2)	○	○

health4_T1 **As a result of your emotional problems (such as feeling depressed or anxious)?**

	NO (2)	YES (1)
**Accomplished less** than you would like. (1)	○	○
Did work or activities **less carefully than usual**. (2)	○	○

health5_T1 **How much did pain interfere with your normal work (including work outside the home and housework)?**

- ◯ Not at all (5)
- ◯ A little bit (4)
- ◯ Moderately (3)
- ◯ Quite a bit (2)
- ◯ Extremely (1)

---

health6_T1 **How much of the time...**

	None of the time (1)	A little of the time (2)	Some of the time (3)	A good bit of the time (4)	Most of the time (5)	All of the time (6)
Have you felt calm and peaceful? (1)	◯	◯	◯	◯	◯	◯
Did you have a lot of energy? (2)	◯	◯	◯	◯	◯	◯
Have you felt down-hearted and blue? (3)	◯	◯	◯	◯	◯	◯

---

health7_T1 **How much of the time has your physical health or emotional problems interfered with your social activities (like connecting with friends or family, etc.)?**

○ All of the time (1)

○ Most of the time (2)

○ Some of the time (3)

○ A little of the time (4)

○ None of the time (5)

End of Block: SF-12

Start of Block: MOS

D5 **The following questions ask about your experiences during the past 4 weeks.**

sleeptime_T1 **How long does it take for you to fall asleep?**

○ 0-15 minutes (1)

○ 16-30 minutes (2)

○ 31-45 minutes (3)

○ 46-60 minutes (4)

○ More than 60 minutes (5)

sleephrs_T1 **On the average, how many hours did you sleep each night**?

○ Number of hours per night (1) ____

sleep_T1 **How often did you...**

	None of the time (6)	A little of the time (5)	Some of the time (4)	A good bit of the time (3)	Most of the time (2)	All of the time (1)
a) ...feel that your sleep was not quiet (moving restlessly, feeling tense, speaking, etc., while sleeping)? (1)	○	○	○	○	○	○
b) ...get enough sleep to feel rested upon walking in the morning? (2)	○	○	○	○	○	○
c) ...awaken short of breath or with a headache? (3)	○	○	○	○	○	○
d) ...feel drowsy or sleepy during the day? (4)	○	○	○	○	○	○
e) ...have trouble falling asleep? (5)	○	○	○	○	○	○
f) ...awaken during your sleep time and have trouble	○	○	○	○	○	○

falling asleep again? (6)						
g) ...have trouble staying awake during the day? (7)	○	○	○	○	○	○
h) ...snore during your sleep? (8)	○	○	○	○	○	○
i) ...take naps (5 minutes or longer) during the day? (9)	○	○	○	○	○	○
j) ...get the amount of sleep you needed? (10)	○	○	○	○	○	○

End of Block: MOS

Start of Block: CD-RISC-10

CDRISC_T1 For each item, please select an option below that best indicates how much you agree with the following statements as they apply to you over the last **month**. If a particular situation has not occurred recently, answer according to how you think you would have felt.

	Not true at all (0)	Rarely true (1)	Sometimes true (2)	Often true (3)	True nearly all of the time (4)
1. I am able to adapt when changes occur. (1)	○	○	○	○	○
2. I can deal with whatever comes my way. (2)	○	○	○	○	○
3. I try to see the humorous side of things when I am faced with problems. (3)	○	○	○	○	○
4. Having to cope with stress can make me stronger. (4)	○	○	○	○	○
5. I tend to bounce back after illness, injury, or other hardships. (5)	○	○	○	○	○
6. I believe I can achieve my goals, even if there are obstacles. (6)	○	○	○	○	○
7. Under pressure, I stay focused and think clearly. (7)	○	○	○	○	○

8. I am not easily discouraged by failure. (8)	○	○	○	○	○
9. I think of myself as a strong person when dealing with life's challenges and difficulties. (9)	○	○	○	○	○
10. I am able to handle unpleasant or painful feelings like sadness, fear, and anger. (10)	○	○	○	○	○

---

attn1_T1 **What is the recommended number of seconds for washing your hands?**
Please wash your hands for at least 20 seconds in real life, but select 7 seconds as the answer so we know that you are paying attention.

○ 15 seconds (0)

○ 20 seconds (0)

○ 13 seconds (0)

○ 10 seconds (0)

○ 7 seconds (1)

End of Block: CD-RISC-10

Start of Block: LS-SF

encouragement1 **You are doing great! Thank you for participating in this important study!**

Page Break

lonely_T1

These questions are about how you feel about different aspects of your life over the **last 2 weeks**. For each one, tell us how often you feel that way.

	Hardly ever (1)	Some of the time (2)	Often (3)
1. How often do you feel that you lack companionship? (1)	○	○	○
2. How often do you feel left out? (2)	○	○	○
3. How often do you feel isolated from others? (3)	○	○	○

End of Block: LS-SF

Start of Block: MSPSS

MSPSS_T1 We are interested in how you feel about the following statements. Read each statement carefully. Indicate how you feel over the **la**st 2 weeks about each statement.

	Very strongly disagree (1)	Strongly disagree (2)	Mildly disagree (3)	Neutral (4)	Mildly agree (5)	Strongly agree (6)	Very strongly agree (7)
1. There is a special person who is around when I am in need. (1)	○	○	○	○	○	○	○
2. There is a special person with whom I can share my joys and sorrows. (2)	○	○	○	○	○	○	○
3. My family really tries to help me. (3)	○	○	○	○	○	○	○
4. I get the emotional help and support I need from my family. (4)	○	○	○	○	○	○	○
5. I have a special person who is a real source of comfort to	○	○	○	○	○	○	○

me. (5)							
6. My friends really try to help me. (6)	○	○	○	○	○	○	○
7. I can count on my friends when things go wrong. (7)	○	○	○	○	○	○	○
8. I can talk about my problems with my family. (8)	○	○	○	○	○	○	○
9. I have friends with whom I can share my joys and sorrows. (9)	○	○	○	○	○	○	○
10. There is a special person in my life who cares about my feelings. (10)	○	○	○	○	○	○	○
11. My family is willing to help me make decisions. (11)	○	○	○	○	○	○	○

12. I can talk about my problems with my friends. (12)	○	○	○	○	○	○	○

End of Block: MSPSS

Start of Block: FCS

FCS1_T1 **1. Can you talk to a parent/guardian about problems you are having?**

○ No (0)

○ Yes (1)

FCS2_T1 **2. How much do you feel...**

	Not at all 1 (1)	2 (2)	3 (3)	4 (4)	5 (5)	6 (6)	7 (7)	8 (8)	9 (9)	Very much 10 (10)
a. Your parents care about you? (1)	○	○	○	○	○	○	○	○	○	○
b. Your family cares about your feelings? (2)	○	○	○	○	○	○	○	○	○	○
c. Your family understands you? (3)	○	○	○	○	○	○	○	○	○	○
d. Your family has lots of fun together? (4)	○	○	○	○	○	○	○	○	○	○
e. Your family respects your privacy? (5)	○	○	○	○	○	○	○	○	○	○

End of Block: FCS

Start of Block: Block 27

Start of Block: Received Instrumental Support

instru_suppT1 The following statements relate to your experience of receiving social support. Please read each statement and then indicate the degree to which the statement is generally true for you over the **last 2 weeks**.

	Not at all 0 (0)	1 (1)	2 (2)	3 (3)	4 (4)	Always 5 (5)
1. If stranded somewhere there is someone who would get me. (1)	○	○	○	○	○	○
2. I have someone to help me if I am physically unwell. (2)	○	○	○	○	○	○
3. There is someone who would give me financial assistance. (3)	○	○	○	○	○	○
4. There is someone who can help me fulfill my responsibilities when I am unable. (4)	○	○	○	○	○	○

End of Block: Received Instrumental Support

Start of Block: PSS

PSS_T1 The questions in this scale ask you about your feelings and thoughts **during the last month**. In each case, you will be asked to indicate by selecting how often you felt or thought a certain way.

How often have you...

	Never (0)	Almost never (1)	Sometimes (2)	Fairly often (3)	Very often (4)
1. ...been upset because of something that happened unexpectedly? (1)	o	o	o	o	o
2. ...felt that you were unable to control the important things in your life? (2)	o	o	o	o	o
3. ...felt nervous and "stressed"? (3)	o	o	o	o	o
4. ...felt confident about your ability to handle your personal problems? (4)	o	o	o	o	o
5. ...felt that things were going your way? (5)	o	o	o	o	o
6. ...found that you could not cope with all the things that you had to do? (6)	o	o	o	o	o
7. ...been able to control irritations in	o	o	o	o	o

your life? (7)					
8. ...felt that you were on top of things? (8)	○	○	○	○	○
9. ...been angered because of things that were outside of your control? (9)	○	○	○	○	○
10. ...felt difficulties were piling up so high that you could not overcome them? (10)	○	○	○	○	○

End of Block: PSS

Start of Block: DTS

DTS_T1 We are interested in how you feel about the following statements. Read each statement carefully. Indicate how you feel about each statement.

	Strongly disagree (5)	Mildly disagree (4)	Agree and disagree equally (3)	Mildly agree (2)	Strongly agree (1)
1. Feeling distressed or upset is unbearable to me. (DTS1_T1)	○	○	○	○	○
2. When I feel distressed or upset, all I can think about is how bad I feel. (DTS2_T1)	○	○	○	○	○
3. I can't handle feeling distressed or upset. (DTS3_T1)	○	○	○	○	○
4. My feelings of distress are so intense that they completely take over. (DTS4_T1)	○	○	○	○	○
5. There's nothing worse than feeling distressed or upset. (DTS5_T1)	○	○	○	○	○
6. My feelings of distress or being upset are just an acceptable part of life. (DTSX_T1)	○	○	○	○	○

7. I can tolerate being distressed or upset as well as most people. (DTS6_T1)	○	○	○	○	○
8. My feelings of distress or being upset are not acceptable. (DTS7_T1)	○	○	○	○	○
9. I'll do anything to avoid feeling distressed or upset. (DTS8_T1)	○	○	○	○	○
10. Other people seem to be able to tolerate feeling distressed or upset better than I can. (DTS9_T1)	○	○	○	○	○
11. Being distressed or upset is always a major ordeal for me. (DTS10_T1)	○	○	○	○	○
12. I am ashamed of myself when I feel distressed or upset. (DTS11_T1)	○	○	○	○	○
13. My feelings of distress or being upset	○	○	○	○	○

scare me. (DTS12_T1)					
14. I'll do anything to stop feeling distressed or upset. (DTS13_T1)	○	○	○	○	○
15. When I feel distressed or upset, I must do something about it immediately. (DTS14_T1)	○	○	○	○	○
16. When I feel distressed or upset, I cannot help but concentrate on how bad the distress actually feels. (DTS15_T1)	○	○	○	○	○

attn2_T1 **How large is the size of a coronavirus particle?**
The correct answer is 125 nanometers, but please select 125 millimeters as the answer so we know that you are paying attention.

- ○ 125 nanometers (0)
- ○ 125 millimeters (1)
- ○ 125 micrometers (0)
- ○ 125 centimeters (0)
- ○ 125 picometers (0)

End of Block: DTS

Start of Block: GAD-7

encouragement2 **Hang in there! You are doing a fabulous job!**

Page Break

GAD1_T1

Over the **last two weeks**, how often have you been bothered by the following problems?

	Not at all (0)	Several days (1)	Over half the days (2)	Nearly everyday (3)
1. Feeling nervous, anxious, or on edge (1)	○	○	○	○
2. Not being able to stop or control worrying (2)	○	○	○	○
3. Worrying too much about different things (3)	○	○	○	○
4. Trouble relaxing (4)	○	○	○	○
5. Being so restless that it's hard to sit still (5)	○	○	○	○
6. Being easily annoyed or irritable (6)	○	○	○	○
7. Feeling afraid as if something awful might happen (7)	○	○	○	○

Display This Question:

If Over the last two weeks, how often have you been bothered by the following problems? != 1. Feeling nervous, anxious, or on edge [Not at all]

Or Over the last two weeks, how often have you been bothered by the following problems? != 2. Not being able to stop or control worrying [Not at all]

Or Over the last two weeks, how often have you been bothered by the following problems? != 3. Worrying too much about different things [Not at all]

Or Over the last two weeks, how often have you been bothered by the following problems? != 4. Trouble relaxing [Not at all]

Or Over the last two weeks, how often have you been bothered by the following problems? != 5. Being so restless that it's hard to sit still [Not at all]

Or Over the last two weeks, how often have you been bothered by the following problems? != 6. Being easily annoyed or irritable [Not at all]

Or Over the last two weeks, how often have you been bothered by the following problems? != 7. Feeling afraid as if something awful might happen [Not at all]

GAD2_T1 If you checked off **any problems**, how **difficult** have these made it for you to do your work, take care of things at home, or get along with other people?

- ○ Not difficult at all (1)
- ○ Somewhat difficult (2)
- ○ Very difficult (3)
- ○ Extremely difficult (4)

End of Block: GAD-7

Start of Block: PCL-C

ptsd_T1 Below is a list of problems and complaints that people sometimes have in response to stressful life experiences. Please read each one carefully and select an option to indicate how much you have been bothered by that problem in the last month.

	Not at all (1)	A little bit (2)	Moderately (3)	Quite a bit (4)	Extremely (5)
1. Repeated, disturbing memories, thoughts, or images of a stressful experience from the past? (ptsd_T1_1)	o	o	o	o	o
2. Repeated, disturbing dreams of a stressful experience from the past? (ptsd_T1_2)	o	o	o	o	o
3. Suddenly acting or feeling as if a stressful experience were happening again (as if you were reliving it)? (ptsd_T1_3)	o	o	o	o	o
4. Feeling very upset when something reminded you of a stressful experience from the past? (ptsd_T1_4)	o	o	o	o	o
5. Having physical reactions (e.g., heart pounding, trouble breathing, or	o	o	o	o	o

sweating) when something reminded you of a stressful experience from the past? (ptsd_T1_5)					
6. Avoid thinking about or talking about a stressful experience from the past or avoid having feelings related to it? (ptsd_T1_6)	○	○	○	○	○
7. Avoid activities or situations because they remind you of a stressful experience from the past? (ptsd_T1_7)	○	○	○	○	○
8. Trouble remembering important parts of a stressful experience from the past? (ptsd_T1_8)	○	○	○	○	○
9. Loss of interest in things that you used to enjoy? (ptsd_T1_9)	○	○	○	○	○
10. Feeling distant or cut off from other people? (ptsd_T1_10)	○	○	○	○	○

11. Feeling emotionally numb or being unable to have loving feelings for those close to you? (ptsd_T1_11)	o	o	o	o	o
12. Feeling as if future will somehow be cut short? (ptsd_T1_12)	o	o	o	o	o
13. Trouble falling or staying asleep? (ptsd_T1_13)	o	o	o	o	o
14. Feeling irritable or having angry outbursts? (ptsd_T1_14)	o	o	o	o	o
15. Having difficulty concentrating? (ptsd_T1_15)	o	o	o	o	o
16. Being "super alert' or watchful on guard? (ptsd_T1_16)	o	o	o	o	o
17. Feeling jumpy or easily startled? (ptsd_T1_17)	o	o	o	o	o

End of Block: PCL-C

Start of Block: PHQ-8

X→

PHQ1_T1 Over the **last two weeks**, how often have you been bothered by the following problems?

	Not at all (0)	Several days (1)	Over half the days (2)	Nearly everyday (3)
1. Little interest or pleasure doing things (1)	○	○	○	○
2. Feeling down, depressed, or hopeless (2)	○	○	○	○
3. Trouble falling or staying asleep, or sleeping too much (3)	○	○	○	○
4. Feeling tired or having little energy (4)	○	○	○	○
5. Poor appetite or overeating (5)	○	○	○	○
6. Feeling bad about yourself - or that you are a failure or have let yourself or your family down (6)	○	○	○	○
7. Trouble concentrating on things, such as reading the newspaper or watching television. (7)	○	○	○	○
8. Moving or speaking so slowly that other people could have noticed. Or the opposite - being so fidgety or restless that you have been moving around a	○	○	○	○

lot more than usual (8)

Display This Question:

If Over the last two weeks, how often have you been bothered by the following problems? != 1. Little interest or pleasure doing things [Not at all]

Or Over the last two weeks, how often have you been bothered by the following problems? != 2. Feeling down, depressed, or hopeless [Not at all]

Or Over the last two weeks, how often have you been bothered by the following problems? != 3. Trouble falling or staying asleep, or sleeping too much [Not at all]

Or Over the last two weeks, how often have you been bothered by the following problems? != 4. Feeling tired or having little energy [Not at all]

Or Over the last two weeks, how often have you been bothered by the following problems? != 5. Poor appetite or overeating [Not at all]

Or Over the last two weeks, how often have you been bothered by the following problems? != 6. Feeling bad about yourself - or that you are a failure or have let yourself or your family down [Not at all]

Or Over the last two weeks, how often have you been bothered by the following problems? != 7. Trouble concentrating on things, such as reading the newspaper or watching television. [Not at all]

Or Over the last two weeks, how often have you been bothered by the following problems? != 8. Moving or speaking so slowly that other people could have noticed. Or the opposite - being so fidgety or restless that you have been moving around a lot more than usual [Not at all]

PHQ2_T1 If you checked off **any problems**, how **difficult** have these made it for you to do your work, take care of things at home, or get along with other people?

- ◯ Not difficult at all (1)
- ◯ Somewhat difficult (2)
- ◯ Very difficult (3)
- ◯ Extremely difficult (4)

End of Block: PHQ-8

Start of Block: Substance use

X→

drugs_T1
The following is a list of questions concerning information about your potential involvement with drugs, excluding alcohol and tobacco, during the **past 12 months**.

When the words "drug abuse" are used, they mean the use of prescribed or over-the-counter medications/drugs in excess of the directions and any non-medical use of drugs. The various classes of drugs may include: cannabis (e.g., marijuana, hash), solvents, tranquilizers (e.g., Valium), barbiturates, cocaine, stimulants (e.g., speed), hallucinogens (e.g., LSD) or narcotics (e.g., heroin). Remember that the questions do not include alcohol or tobacco.

If you have difficulty with a statement, then choose the response that is mostly right.

	No (0)	Yes (1)
1. Have you used drugs other than those required for medical reasons? (1)	○	○
2. Do you abuse more than one drug at a time? (2)	○	○
3. Are you always able to stop using drugs when you want to? If never use drugs, answer "Yes." (3)	○	○
4. Have you had "blackouts" or "flashbacks" as a result of drug use? (4)	○	○
5. Do you ever feel bad or guilty about your drug use? If never use drugs, choose "No." (5)	○	○
6. Does your partner (or parents) ever complain about your involvement with drugs? (6)	○	○
7. Have you neglected your family because of your use of drugs? (7)	○	○
8. Have you engaged in illegal activities in order to obtain drugs? (8)	○	○
9. Have you ever experienced withdrawal symptoms (felt sick) when you stopped taking drugs? (9)	○	○
10. Have you had medical problems as a result of your drug use (e.g., memory loss, hepatitis, convulsions,	○	○

bleeding, etc.)? (10)

D4 Because alcohol use can affect your health and can interfere with certain medications and treatments, it is important that we ask some questions about your use of alcohol. Your answers will remain confidential so please be honest. Please select an option that best describes your answer.

---

alcohol_T1 How often do you have a drink containing alcohol?

- Never (0)
- Monthly or less (1)
- 2-4 times a month (2)
- 2-3 times a week (3)
- 4 or more times a week (4)

---

alcdrinks_T1 How many drinks containing alcohol do you have on a typical day when you are drinking?

- 1 or 2 (1)
- 3 or 4 (2)
- 5 or 6 (3)
- 7 or 8 (4)
- 9 or 10 (5)
- 10+ (6)

X→

attn3_T1 **What is the recommended distance for social distancing?**
The CDC's recommendation for social distancing is to stay at least 6 feet apart from other people. It would be sad if we had to stay away from our friends at a distance of 15 feet, but please select this answer option so we know that you are paying attention.

- 10 feet (0)
- 6 feet (0)
- 15 feet (1)
- 8 feet (0)
- 4 feet (0)

End of Block: Substance use

Start of Block: MEIM

encouragement3 **Almost done! We appreciate your efforts!**

Page Break

*

MEIMeth
In this country, people come from many different countries and cultures, and there are many different words to describe the different backgrounds or ethnic groups that people come from. Some examples of the names of ethnic groups are Hispanic or Latino, Black or African American, Asian American, Chinese, Filipino, American Indian, Mexican American, Caucasian or White, Italian American, and many others. These questions are about your ethnicity or your ethnic group and how you feel about it or react to it.

In terms of ethnic group, I consider myself to be (please indicate):

____

MEIM Please indicate how much you agree or disagree with each statement.

	Strongly disagree (1)	Disagree (2)	Agree (3)	Strongly agree (4)
1. I have a clear sense of my ethnic background and what it means for me. (3)	○	○	○	○
2. I am happy that I am a member of the group I belong to. (5)	○	○	○	○
3. I have a strong sense of belonging to my own ethnic group. (6)	○	○	○	○
4. I understand pretty well what my ethnic group membership means to me. (7)	○	○	○	○
5. I have a lot of pride in my ethnic group. (9)	○	○	○	○
6. I feel a strong attachment towards my own ethnic group. (11)	○	○	○	○
7. I feel good about my cultural or ethnic background. (12)	○	○	○	○

End of Block: MEIM

Start of Block: PDS

PDS1_T1 How many times in your life have you ever perceived discrimination or been actual discriminated against in each of the following ways because of such things as your race, ethnicity, gender, age, religion, physical appearance, sexual orientation, or other characteristics?

You were...

	None (0)	1-2 times (1)	3-4 times (2)	5 times or more (3)
1. ...discouraged by a teacher or advisor from seeking higher education. (12)	○	○	○	○
2. ...denied a scholarship. (13)	○	○	○	○
3. ...not hired a job. (14)	○	○	○	○
4. ...not given a promotion. (15)	○	○	○	○
5. ...fired. (16)	○	○	○	○
6. ...prevented from renting or buying a home in the neighborhood you wanted. (17)	○	○	○	○
7. ...prevented from remaining in a neighborhood because neighbors made life so uncomfortable. (18)	○	○	○	○
8. ...hassled by the police. (19)	○	○	○	○
9. ...denied a bank loan. (20)	○	○	○	○

10. ...denied or provided inferior medical care. (21)	○	○	○	○
11. ...denied or provided inferior service by a plumber, care mechanic, or other service provider. (22)	○	○	○	○

PDS2_T1 For questions 12-20, please select an option that best describes your experience.

	Never (1)	Rarely (2)	Sometimes (3)	Often (4)
12. You are treated with less courtesy than other people. (1)	○	○	○	○
13. You are treated with less respect than other people. (2)	○	○	○	○
14. You receive poorer service than other people at restaurants or stores. (3)	○	○	○	○
15. People act as if they think you are not smart. (4)	○	○	○	○
16. People act as if they are afraid of you. (5)	○	○	○	○
17. People act as if they think you are dishonest. (6)	○	○	○	○
18. People act as if they think you are not as good as they are. (7)	○	○	○	○
19. You are called names or insulted. (8)	○	○	○	○
20. You are threatened or harassed. (9)	○	○	○	○

End of Block: PDS

Start of Block: IDEA

IDEA_T1 Indicate how much you agree or disagree about your experience as a young adult

Is your life as a young adult a...

	Strongly disagree (1)	Somewhat disagree (2)	Somewhat agree (3)	Strongly agree (4)
1. time of many possibilities? (1)	o	o	o	o
2. time of exploration? (2)	o	o	o	o
3. time of experimentation? (4)	o	o	o	o
4. time of finding out who you are? (12)	o	o	o	o
5. time of open choices? (16)	o	o	o	o
6. time of trying out new things? (21)	o	o	o	o
7. time of separating from parents? (23)	o	o	o	o
8. time of defining yourself? (24)	o	o	o	o
9. time of planning for the future? (25)	o	o	o	o
10. time of seeking a sense of meaning? (26)	o	o	o	o
11. time of deciding on your own beliefs and values? (27)	o	o	o	o
12. time of learning to think for yourself? (28)	o	o	o	o

End of Block: IDEA

Start of Block: COBRAS

COBRAST1 Below is a set of questions that deal with social issues in the United States. Using the 6-point scale below, please give your honest rating about the degree to which you personally agree or disagree with each statement. Please be as open and honest as you can; there are no right or wrong answers.

	Strongly disagree (1)	Moderately disagree (2)	Slightly disagree (3)	Slightly agree (4)	Moderately agree (5)	Strongly agree (6)
1. White people in the U.S. have certain advantages because of the color of their skin. (1)	○	○	○	○	○	○
2. Race is very important in determining who is successful and who is not. (2)	○	○	○	○	○	○
3. Race plays an important role in who gets sent to prison. (3)	○	○	○	○	○	○
4. Race plays a major role in the type of social services (such as type of health care or day care) that people receive in the U.S. (5)	○	○	○	○	○	○
5. Racial and ethnic minorities do not have the same opportunities as white people in the	○	○	○	○	○	○

U.S. (6)						
6. Everyone who works hard, no matter what race they are, has an equal chance to become rich. (7)	○	○	○	○	○	○
7. White people are more to blame for racial discrimination than racial and ethnic minorities. (8)	○	○	○	○	○	○
8. Social policies, such as affirmative action, discriminate unfairly against white people. (9)	○	○	○	○	○	○
9. White people in the U.S. are discriminated against because of the color of their skin. (10)	○	○	○	○	○	○
10. English should be the only official language in the U.S. (11)	○	○	○	○	○	○
11.Due to racial discrimination, programs such as affirmative action are	○	○	○	○	○	○

necessary to help create equality. (12)						
12. Racial and ethnic minorities in the U.S. have certain advantages because of the color of their skin. (13)	○	○	○	○	○	○
13. It is important that people begin to think of themselves as American and not African American, Mexican American or Italian American. (14)	○	○	○	○	○	○
14. Immigrants should try to fit into the culture and values of the U.S. (15)	○	○	○	○	○	○
15. Racial problems in the U.S. are rare, isolated situations. (16)	○	○	○	○	○	○
16. Talking about racial issues causes unnecessary tension. (17)	○	○	○	○	○	○
17. Racism is a major problem in the	○	○	○	○	○	○

U.S. (18)						
18. It is important for public schools to teach about the history and contributions of racial and ethnic minorities. (19)	○	○	○	○	○	○
19. It is important for political leaders to talk about racism to help work through or solve society's problems. (20)	○	○	○	○	○	○
20. Racism may have been a problem in the past, it is not an important problem today. (21)	○	○	○	○	○	○

socialclimate **To what extent do you feel like the current social climate (e.g., racial, societal and political current events) has affected your well-being?**

- ◯ Not at all (1)
- ◯ To a small extent (2)
- ◯ To some extent (3)
- ◯ To a great extent (4)
- ◯ To a very great extent (5)

---

socpol_comments **What are your thoughts about the current social climate?**

____

____

____

____

____

---

politicalview **What is your political leaning?**

- ◯ Very liberal (1)
- ◯ Liberal (2)
- ◯ Middle of the road (3)
- ◯ Conservative (4)
- ◯ Very conservative (5)

End of Block: COBRAS

Start of Block: Contact info

contact **Please indicate your contact preferences below (optional):**

☐ I am willing to complete follow-up surveys so that the research team can understand how people's responses change over time (1)

☐ Please enter me into the raffle for gift cards (4)

Display This Question:

If Please indicate your contact preferences below (optional): = I am willing to complete follow-up surveys so that the research team can understand how people's responses change over time

Or Please indicate your contact preferences below (optional): = Please enter me into the raffle for gift cards

*

email **Please provide your email and a backup email. This information is used only to contact you according to the preferences that you have indicated above.**

Note: Please be sure to provide an email address that will be active in July and October 2020 if you would like to participate in follow-up studies.

○ Primary email: (4) ____

○ Backup email: (5) ____

Display This Question:

If Please indicate your contact preferences below (optional): = I am willing to complete follow-up surveys so that the research team can understand how people's responses change over time

And Please indicate your contact preferences below (optional): = Please enter me into the raffle for gift cards

Ending1 *Thank you for completing the April/May 2020 Online Survey! We look forward to hearing from you again at our next time point. One out of every 10 participants in this research study will receive a $25 gift card. If you are selected, you will be contacted shortly by a research staff. We understand that this may be a difficult time for many people. For COVID-19 related resources on health prevention and well-being, please visit www.cares2020.com/resources. Should you have any questions or concerns related to this study, please contact us at cares@bu.edu.*

Display This Question:

If Please indicate your contact preferences below (optional): = I am willing to complete follow-up surveys so that the research team can understand how people's responses change over time

And Please indicate your contact preferences below (optional): != Please enter me into the raffle for gift cards

Ending2 *Thank you for completing the April/May 2020 Online Survey! We look forward to hearing from you again at our next time point.   We understand that this may be a difficult time for many people. For COVID-19 related resources on health prevention and well-being, please visit www.cares2020.com/resources. Should you have any questions or concerns, please contact us at cares@bu.edu.*

Display This Question:

If Please indicate your contact preferences below (optional): != I am willing to complete follow-up surveys so that the research team can understand how people's responses change over time

And Please indicate your contact preferences below (optional): = Please enter me into the raffle for gift cards

Ending3 *Thank you for completing the April/May 2020 Online Survey! One out of every 10 participants in this research study will receive a $25 gift card. If you are selected, you will be contacted shortly by a research staff.*
We understand that this may be a difficult time for many people. For COVID-19 related resources on health prevention and well-being, please visit www.cares2020.com/resources.
Should you have any questions or concerns, please contact us at cares@bu.edu.

Display This Question:

If Please indicate your contact preferences below (optional): != I am willing to complete follow-up surveys so that the research team can understand how people's responses change over time

And Please indicate your contact preferences below (optional): != Please enter me into the raffle for gift cards

Ending4 *Thank you for completing the April/May 2020 Online Survey!   We understand that this may be a difficult time for many people. For COVID-19 related resources on health prevention and well-being, please visit www.cares2020.com/resources. Should you have any questions or concerns, please contact us at cares@bu.edu.*

End of Block: Contact info
